# ETHYLENE RESPONSE FACTORS 4.1/4.2 with an EAR motif repress anthocyanin biosynthesis in red-skinned pears

**DOI:** 10.1093/plphys/kiad068

**Published:** 2023-02-03

**Authors:** Hongye Sun, Kangdi Hu, Shuwei Wei, Gaifang Yao, Hua Zhang

**Affiliations:** School of Food and Biological Engineering, Hefei University of Technology, Hefei 230009, China; School of Food and Biological Engineering, Hefei University of Technology, Hefei 230009, China; Shandong Institute of Pomology, Tai’an 271000, China; School of Food and Biological Engineering, Hefei University of Technology, Hefei 230009, China; School of Food and Biological Engineering, Hefei University of Technology, Hefei 230009, China

## Abstract

Red-skinned pears (*Pyrus* L.) are preferred to consumers for their attractive color and abundant anthocyanins. Pyrus ETHYLENE RESPONSE FACTOR 3 (PyERF3) positively regulates anthocyanin biosynthesis through interacting with Pyrus myeloblastosis family 114 (PyMYB114) and Pyrus basic helix-loop-helix 3 (PybHLH3) in red-skinned pears. However, the role of APETALA2/ethylene response factors (AP2/ERFs), which negatively regulate anthocyanin biosynthesis, remains unclear in red-skinned pears. Here, we validated that 2 AP2/ERFs, PyERF4.1 and PyERF4.2, screened from the transcriptome data of ‘Starkrimson’ pear (*Pyrus communis* L.) and its green mutant, inhibit anthocyanin biosynthesis in transgenic pear calli, as well as in overexpression and gene-edited tomato (*Solanum lycopersicum*) fruits. Meanwhile, the co-transformation of *PyERF4.1*/*PyERF4.2* with *PyERF3*–*PyMYB114*–*PybHLH3* inhibited anthocyanin biosynthesis in pear fruits and strawberry (*Fragaria vesca*) receptacles. Further assays showed that PyMYB114 activated the transcription of *PyERF4.1*/*PyERF4.2*; PyERF4.1/PyERF4.2 then interacted with PyERF3 to affect the stability of the PyERF3–PyMYB114–PybHLH3 complex, thereby inhibiting the transcription of the anthocyanin biosynthesis gene *Pyrus anthocyanidin synthase* (*PyANS*). Furthermore, deletion of the ERF-associated-amphiphilic repression (EAR) motif eliminated the inhibitory effect of PyERF4.1/PyERF4.2 on anthocyanin biosynthesis, and a mutation of the PyERF4.2-EAR motif (LxLxM to LxLxL) strengthened the inhibitory effect, demonstrating that the EAR motif is indispensable for the inhibitory effect of PyERF4.1/PyERF4.2 on anthocyanin biosynthesis in pears. Our study has shed light on a feedback regulatory loop mechanism that balances the excessive accumulation of anthocyanins in red-skinned pears, providing insights into the regulatory mechanism of anthocyanin biosynthesis and the regulatory network of coloration in red-skinned pears.

## Teaser

A regulatory feedback loop mechanism involving repressor ETHYLENE RESPONSE FACTORS 4.1/4.2 balances anthocyanin overaccumulation in red-skinned pears.

## Introduction

Pear (*Pyrus* L.) is among the most popular worldwide fruits, and red-skinned pears are preferred to consumers for their attractive color and abundant anthocyanins (Li et al. 2020). Anthocyanins are widely found in plants, and they not only enhance the dissemination of plants, but also have a major role in stress responses, such as preventing plants from ultraviolet damage, scavenging free radicals, and increasing antioxidant activity (Peng et al. 2017). Furthermore, anthocyanins may also play an important role on humans against neurological and cardiovascular diseases (Yang et al. 2017).

Anthocyanins are biosynthesized by the flavonoid pathway (Koes et al. 2005; Hichri et al. 2011), where phenylalanine ammonia-lyase (PAL), chalcone synthase (CHS), chalcone isomerase (CHI), flavanone-3-hydroxylase (F3H), dihydroflavonol 4-reductase (DFR), anthocyanidin synthase (ANS), and UDP-glucose: flavonoid 3-glucosyl transferase (UFGT) are key enzymes (Tanaka et al. 2008; Zhao and Dixon 2009). Many transcription factors (TFs) are involved in regulating plant anthocyanin biosynthesis. Myeloblastosis family (MYB) TFs, basic helix-loop-helix (bHLH) TFs, and tryptophan-aspartic acid repeat (WDR) TFs regulated anthocyanin biosynthesis in different plants (Hichri et al. 2010; Xu et al. 2014). In general, anthocyanins are controlled mainly by the MYB–bHLH–WD40 (MBW) complex which composed of at least 3 TFs, belonging to 3 different families (Ramsay and Glover 2005; Liu et al. 2021). MYBs, as central regulators in the MBW complex, have been widely reported to regulate anthocyanin biosynthesis individually or together with bHLHs in horticultural plants. For example, overexpression of *FvMYB10* in strawberry (*Fragaria vesca*) and *SlMYB75* in tomato (*Solanum lycopersicum*) fruits substantially promoted anthocyanin biosynthesis (Jian et al. 2019; Castillejo et al. 2020). In apple (*Malus domestica*), MdMYB10 enhances anthocyanin synthesis by interacting with MdbHLH3 and MdbHLH33 (Espley et al. 2007). In pear, PyMYB114, PyMYB10 or PyMYB10b interacted with PybHLH3 and substantially enhanced anthocyanin biosynthesis (Zhai et al. 2016; Yao et al. 2017). Moreover, peach (*Prunus persica*) PpMYB18 and red-skinned pear PyMYB140 act as repressors competing with anthocyanin-associated MYB activators to bind bHLHs for regulating anthocyanin accumulation (Zhou et al. 2019; Ni et al. 2021).

There is increasing evidence that in addition to the MBW complex, the APETALA2/ethylene response factor (AP2/ERF) family also participates in regulating anthocyanin biosynthesis (An et al. 2020a, 2020b; Ma et al. 2021). AP2/ERFs are a large family of plant-specific TFs with a conserved AP2 domain and have been found to be involved in plant developmental processes, like regulating plant secondary metabolite synthesis, and various stress responses via binding to the short cis-acting elements such as the GCC-box and dehydration responsive elements/C-repeat element motifs in the target gene promoter (Liu et al. 1998; Fujimoto et al. 2000; Li et al. 2018). Generally, ERF proteins have no specific sequence motifs as transcriptional activators but tend to be enriched in acidic amino acids; however, as repressors, their C-terminal region usually contain an ERF-associated-amphiphilic repression (EAR) motif defined by the consistent sequence patterns of either LxLxL or DLNLxP (Ohta et al. 2001; Li et al. 2018; Yang et al. 2018). EAR motif-mediated transcriptional repression is the major form of transcriptional repression found in plants to date (Kagale et al. 2010; Choi et al. 2018).

A number of studies have revealed that AP2/ERFs participate in regulating anthocyanin biosynthesis by interacting with the MBW complex, and they have diverse regulatory models of anthocyanin biosynthesis in different plant species under different conditions (Yao et al. 2017; An et al. 2018; Wu et al. 2020; Ni et al. 2021). In phytohormone-induced anthocyanin biosynthesis, MdERF1B and MdERF3 directly activate the expression of *MdMYB11* and *MdMYB1*, respectively, thereby promoting ethylene-induced anthocyanin biosynthesis in apple fruits (An et al. 2018; Zhang et al. 2018); jasmonate and ethylene-regulated PyERF22 enhances the activation of the *PyUFGT* promoter by interacting with PyMYB10 and PyMYB10b to promote lanolin-induced anthocyanin biosynthesis in ‘Zaosu’ pear fruits (*Pyrus pyrifolia* Nakai) (Wu et al. 2020); ethylene-activated PyERF105 induces the repressor *PyMYB140* expression for inhibiting anthocyanin biosynthesis in red-skinned pear fruits (Ni et al. 2021). In light-induced anthocyanin biosynthesis, Py4ERF24 and Py12ERF96 are verified to enhance blue light-induced anthocyanin biosynthesis by interacting with PyMYB114 in ‘Hongzaosu’ pear fruits (*P. pyrifolia* Nakai) (Ni et al. 2019). In drought stress-induced anthocyanin biosynthesis, MdERF38 interacts with MdMYB1 to promote drought stress-induced anthocyanin biosynthesis in apple fruits (An et al. 2020b). Besides, PyERF3 screened from ‘Starkrimson’ pear (*P. communis* L.) and its green mutant interacts with PyMYB114 and PybHLH3 to regulate anthocyanin biosynthesis (Yang et al. 2015; Yao et al. 2017). Thus, the above studies showed that many AP2/ERFs positively regulate anthocyanin biosynthesis, whereas a negative regulation of anthocyanin biosynthesis by repressor-type AP2/ERFs in horticultural plants is unclear. Meanwhile, the color of red-skinned pears is often unstable and sometimes does not develop, which seriously restricts the development of red-skinned pear industry (Ni et al. 2021). Hence, it is of great importance and necessity to investigate how AP2/ERFs inhibit anthocyanin biosynthesis and thus balance the excessive accumulation of anthocyanins in red-skinned pears.

In this study, 2 *AP2/ERFs*, *PyERF4.1* and *PyERF4.2*, containing EAR motifs were screened from the transcriptome data of ‘Starkrimson’ pear and its green mutant during 3 fruit developmental stages by bioinformatics and correlation analysis. The inhibitory function of PyERF4.1 and PyERF4.2 on anthocyanin biosynthesis was verified in transgenic pear calli, stable overexpressed tomato fruits, and deletion mutation tomato fruits obtained by clustered regularly interspaced short palindromic repeats/CRISPR associated nuclease 9 (CRISPR/Cas9) editing. Co-expression of *PyERF4.1*/*PyERF4.2* with *PyERF3*–*PyMYB114*–*PybHLH3* in strawberry receptacles and pears by the transient expression system substantially reduced anthocyanins accumulation. Further analysis showed that PyMYB114 binds to the *PyERF4.1* or *PyERF4.2* promoters and activates their transcription, then PyERF4.1 and PyERF4.2 were verified to interact with PyERF3 through EAR motif and this interaction results in decreased stability of the PybHLH3–PyMYB114–PyERF3 complex, thereby repressing the anthocyanin biosynthesis gene *Pyrus anthocyanidin synthase* (*PyANS*) transcription. Besides, the integrity of the EAR motif of PyERF4.1 and PyERF4.2 determines the strength of the inhibitory effect. Therefore, our data provide insights into the inhibitory mechanism of PyERF4.1 and PyERF4.2 for anthocyanin biosynthesis in red-skinned pears, which could help to refine the network of regulation for anthocyanin biosynthesis in pears.

## Results

### Screening of AP2/ERF candidate genes by transcriptome data and bioinformatics analysis

**Figure 1.**
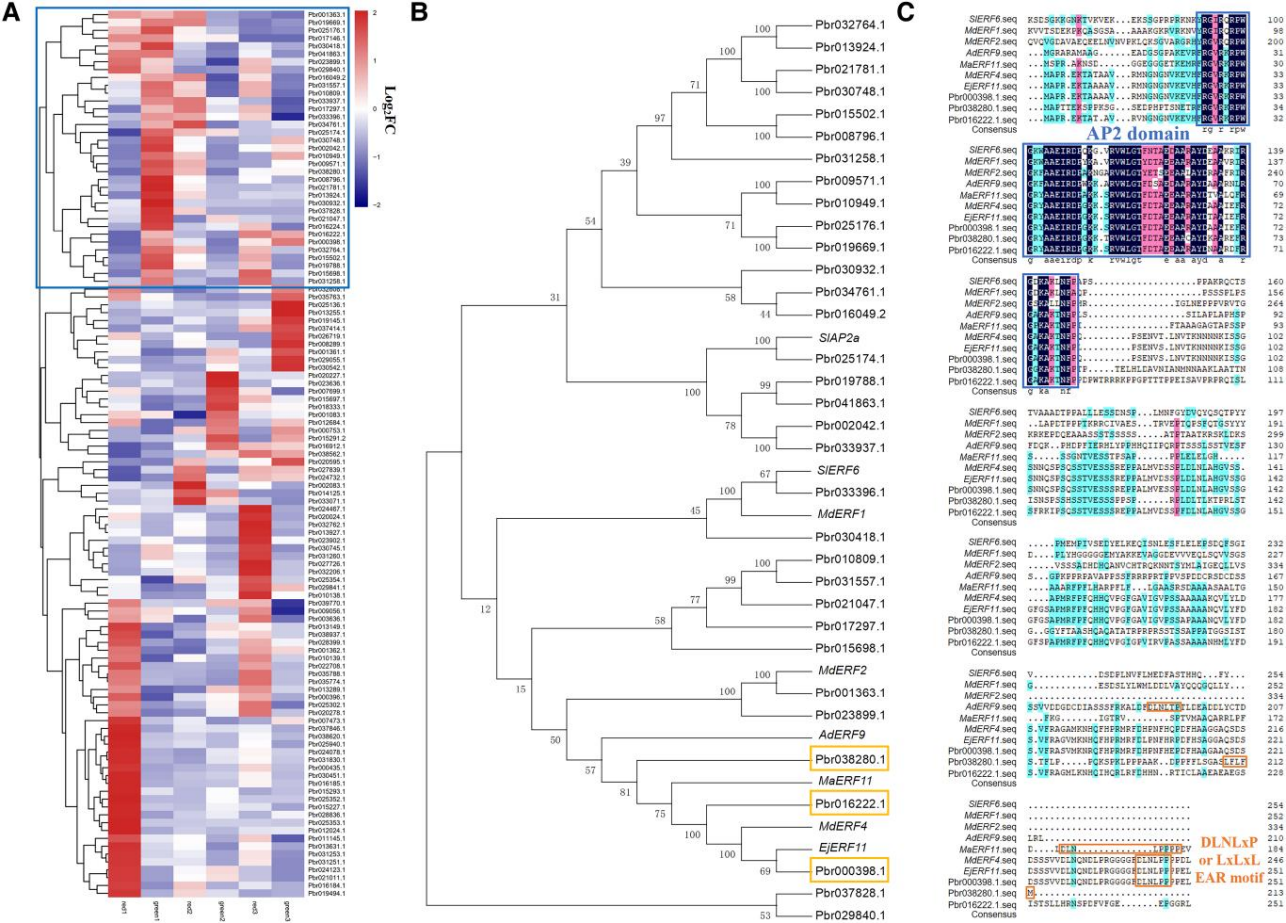
The candidate *AP2/ERF* genes screened from transcriptome data of ‘Starkrimson’ pear and its green mutant bioinformatics analysis. A) Heat map of 114 AP2/ERF genes differentially expressed at different developmental stages in the peel of ‘Starkrimson’ pear and its green mutant. Red1, red2, and red3 are the samples of ‘Starkrimson’ red-skinned pear at 40, 55 and 85 DAFB; green1, green2, and green3 denote the samples of the green mutant of ‘Starkrimson’ at 40, 55 and 85 DAFB, respectively. The value (Log_2_FC) of each ERF gene was calculated by comparing the expression of each gene in ‘Starkrimson’ pear to its green mutant. The square box shows the AP2/ERFs screened for downregulated expression in red pears. B) The downregulated expression of 41 AP2/ERFs in different plant species are analyzed in the phylogenetic tree. The C-terminus of the ERF domain was analyzed by the MEGA7 Program using the neighbor-joining method. The figures next to the branches indicated the percentage of replicated trees in which the relevant taxa clustered together in the bootstrap test (1,000 replications). The yellow boxes show Pbr000398.1, Pbr038280.1, Pbr016222.1, respectively. C) The amino acid sequence alignment analysis of the candidate AP2/ERFs. EAR motif, ERF-associated-amphiphilic repression motif. The blue box shows the protein sequence of AP2 domain, and the orange box shows the protein sequence and location of the EAR negative repressor motif.

To explore the regulatory mechanism of anthocyanin biosynthesis in red-skinned pears by AP2/ERFs, deep exploration of transcriptome data of red-skinned pear ‘Starkrimson’ and its green mutant revealed 114 *AP2/ERFs* were differentially expressed genes at 40, 55, and 85 days after full bloom (DAFB) (Yang et al. 2015). Among of them, 33 *AP2/ERFs* were downregulated in ‘Starkrimson’ pear comparing with its green mutant by heat map analysis (Fig. 1A). Next, 33 *AP2/ERFs* were compared with the reported negative regulators associated with color changes in other species (SlAP2a, SlERF6, MdERF1, MdERF2, AdERF9, MaERF11, MdERF4, and EjERF11) and a phylogenetic tree was constructed using MEGA7 software and the neighbor-joining method with bootstrap analysis (1,000 replicates). As shown in Fig. 1B, Pbr000398.1, Pbr038280.1, and Pbr016222.1 were most closely related to the evolution of color change-related negative regulators EjERF11, MdERF4, MaERF11, and AdERF9, and expression of their genes were substantially downregulated during the critical period of red-skinned pear peel coloration, so Pbr000398.1, Pbr038280.1, and Pbr016222.1 were selected for the further study.

Furthermore, amino acid sequence alignment showed the presence of the AP2 domain in all these proteins, and it was found that Pbr000398.1, EjERF11, MdERF4, MaERF11, and AdERF9 contained an intact complete EAR motif (DLNLxP), whereas Pbr038280.1 had an incomplete EAR motif with a mutated amino acid (LxLxM), Pbr016222.1 had no EAR motif (Fig. 1C). Thus, it could be hypothesized that Pbr000398.1 and Pbr038280.1 negatively regulate anthocyanin biosynthesis in red-skinned pears, and the expression levels of their genes were substantially downregulated in ‘Starkrimson’ pears compared with the green mutant at different developmental stages. Therefore, Pbr000398.1 and Pbr038280.1 were screened for further investigation and named PyERF4.1 and PyERF4.2, respectively.

### Negative correlation of *PyERF4.1*/*PyERF4.2* expression with anthocyanin biosynthesis in red-skinned pears

**Figure 2.**
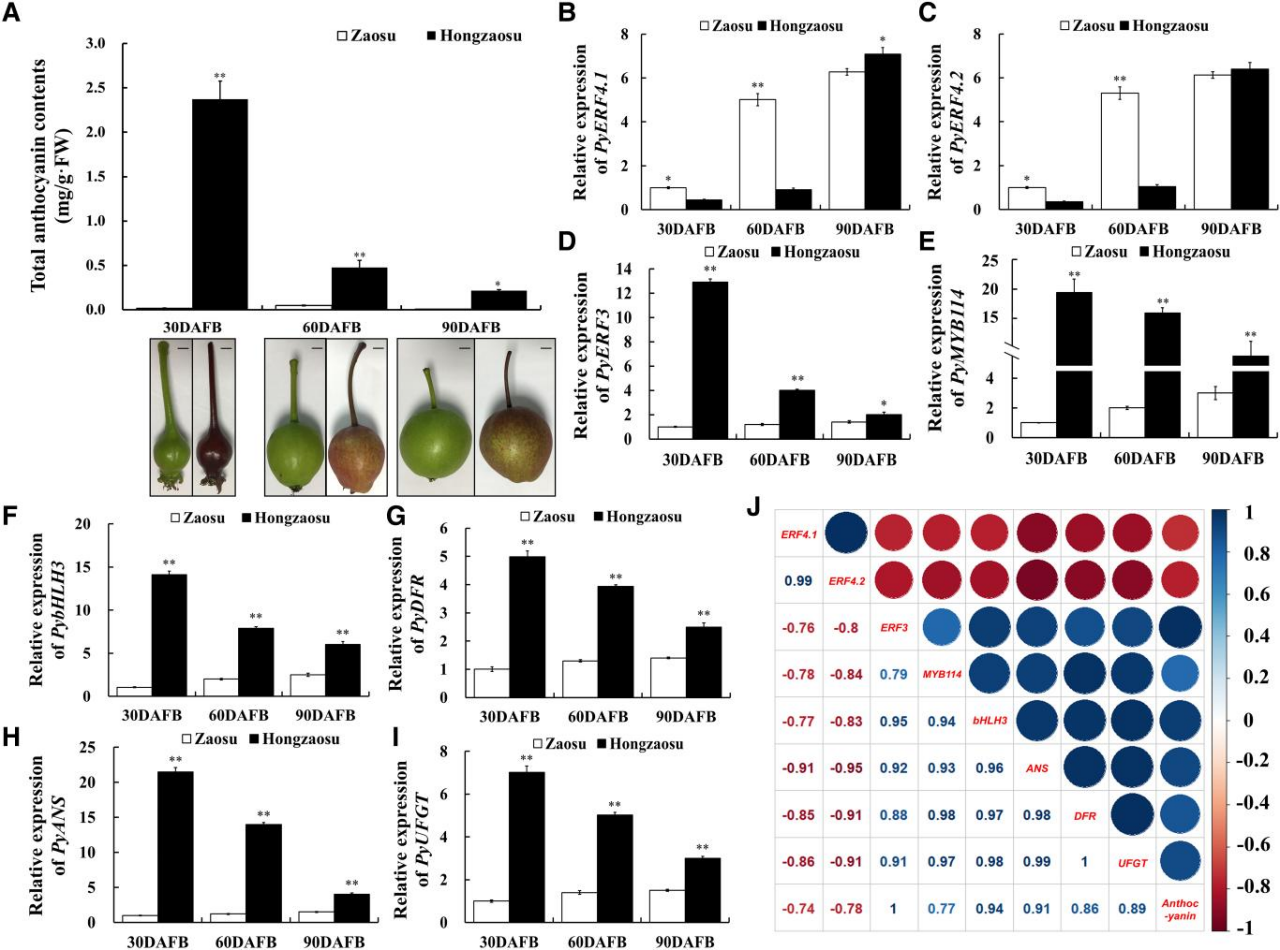
Assessing the correlation of candidate AP2/ERFs with anthocyanin biosynthesis-related genes in ‘Zaosu’ and ‘Hongzaosu’ pear cultivars. A) The appearance and total anthocyanin contents of ‘Zaosu’ and ‘Hongzaosu’ pears at 30, 60 and 90 DAFB. DAFB, days after full bloom. Scale bars = 1 cm. B–F) Relative expression of *PyERF4.1*, *PyERF4.2*, *PyERF3*, *PyMYB114*, and *PybHLH3* in ‘Zaosu’ and ‘Hongzaosu’ pears at 30, 60 and 90 DAFB. G–I) Relative expression of *PyDFR*, *PyANS*, and *PyUFGT* in ‘Zaosu’ and ‘Hongzaosu’ pears at 30, 60 and 90 DAFB. J) Correlation analysis among the anthocyanin contents and gene expression levels of *PyERF4.1*, *PyERF4.2*, *PyERF3*, *PyMYB114*, *PybHLH3*, *PyDFR*, *PyANS*, and *PyUFGT*. R scripts were used to analyze Pearson's correlation coefficients. “+” represents a positive correlation, “−” represents a negative correlation. Larger size and darker color of the circles mean stronger correlation. Bars indicate mean values ± SD from 3 biological replicates. Statistical analysis was carried out with Student's *t*-test, and significance was marked with asterisks (*P < 0.05) or (**P < 0.01).

For exploring and verifying associations of *PyERF4.1*/*PyERF4.2* with the structural genes and TFs involved in anthocyanin biosynthesis, the anthocyanin content of red-skinned pears ‘Hongzaosu’, ‘Starkrimson’, and green-skinned pears ‘Zaosu’, ‘Jinzheng NO.1’ were measured at 30, 60 and 90 DAFB. The results showed that total anthocyanin contents of ‘Hongzaosu’ and ‘Starkrimson’ pear peels were the highest at 30 DAFB, then decreased with fruit development, and they were always higher than those of ‘Zaosu’ and ‘Jinzheng NO.1’ pear peels (*P* < 0.01) (Fig. 2A; Supplemental Fig. S1A). In addition, *PyERF4.1* and *PyERF4.2* expression levels were much higher in ‘Zaosu’ and ‘Jinzheng NO.1’ pear peels than in ‘Hongzaosu’ and ‘Starkrimson’ pear peels and increased with fruit development, while *PyERF3*, *PyMYB114*, *PybHLH3*, *PyDFR*, *PyANS*, and *PyUFGT* expression levels showed opposite patterns (Fig. 2, B–I; Supplemental Fig. S1, B–I). The correlation analysis showed that *PyERF4.1* and *PyERF4.2* expression levels were substantially negatively correlated with anthocyanins content, the expression levels of *PyERF3*, *PyMYB114*, *PybHLH3*, and the expression levels of anthocyanin biosynthesis genes *PyDFR*, *PyANS* and *PyUFGT* (Fig. 2J). This suggested that PyERF4.1 and PyERF4.2 may negatively regulate anthocyanin biosynthesis in red-skinned pears.

### PyERF4.1 and PyERF4.2 negatively regulate anthocyanin biosynthesis in pears

**Figure 3.**
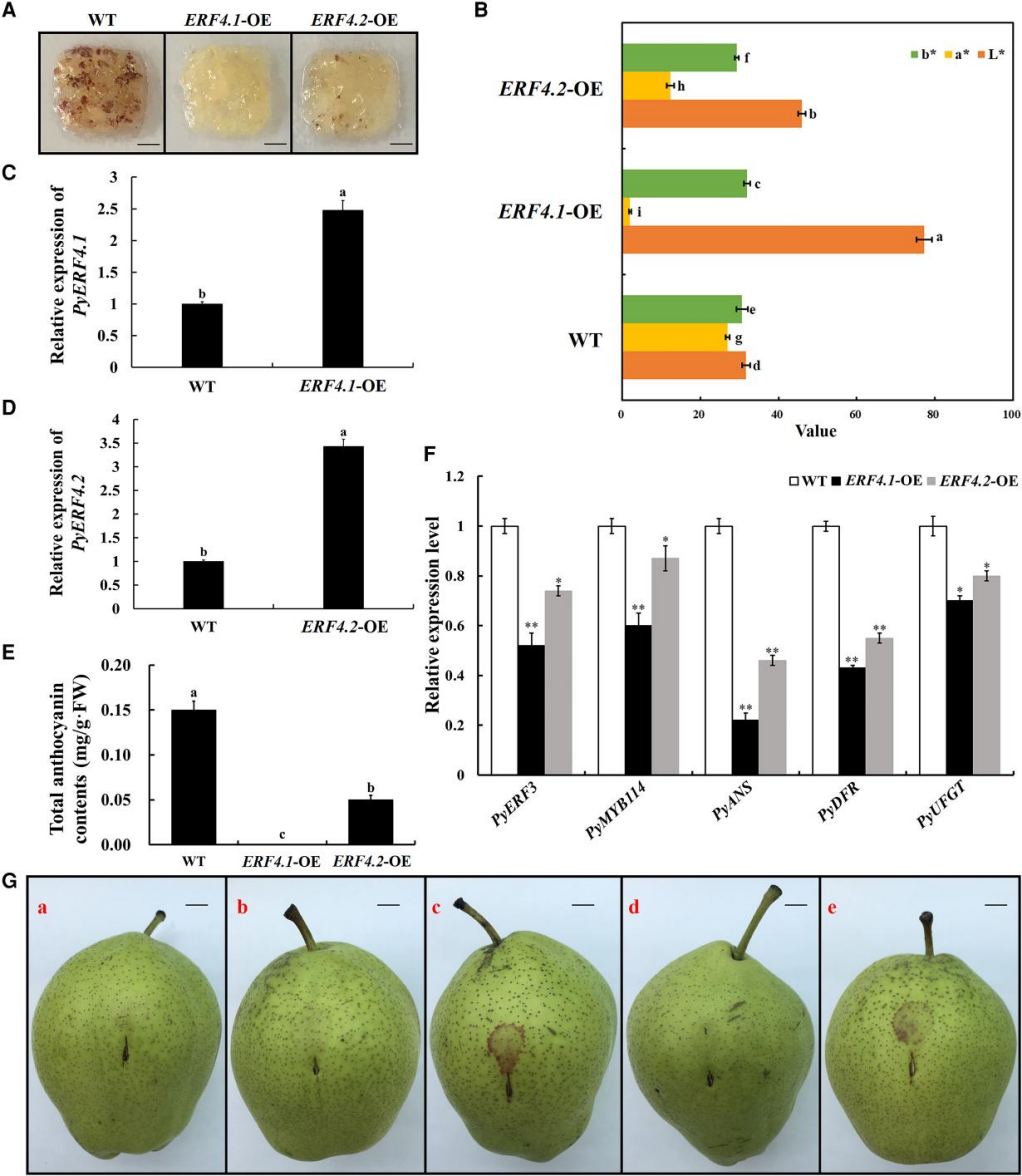

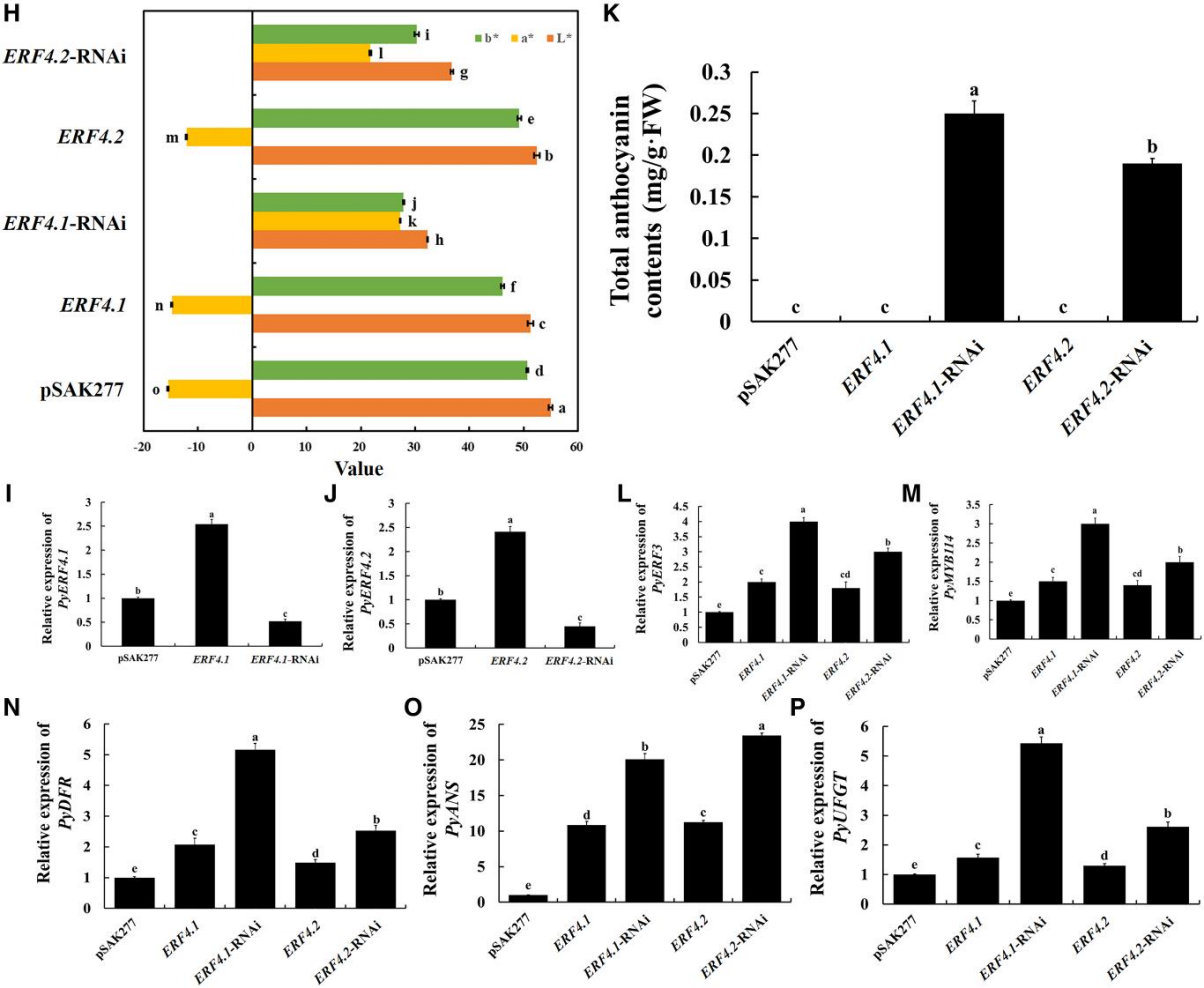
Validation of the inhibitory effect of PyERF4.1 and PyERF4.2 on anthocyanin biosynthesis in pears. A) The phenotype of *PyERF4.1* and *PyERF4.2* overexpressed pear calli after 15 d of the light treatment. WT, wild type; OE, overexpression. Scale bars = 2 mm. B) The difference in color is indicated by the values of L*, a*, and b*. L* indicates luminance; a* indicates a range from green to magenta; b* indicates a range from yellow to blue. Bars indicate mean values ± SD from 6 biological replicates. C, D) Relative expression of *PyERF4.1* and *PyERF4.2* in transgenic pear calli. Data are presented as means ± SD (*n* = 3). E) Total anthocyanin contents of *PyERF4.1* and *PyERF4.2* overexpressed in pear calli. Bars indicate mean values ± SD from 3 biological replicates. F) Relative expression of *PyERF3*, *PyMYB114*, *PyDFR*, *PyANS*, and *PyUFGT* in transgenic pear calli. Data are presented as means ± SD (*n* = 3). G) The phenotype of ‘Zaosu’ pear peels after infiltration with RNAi-induced gene silencing method: a, pSAK277; b, *PyERF4.1;* c, *PyERF4.1*-RNAi; d, *PyERF4.2*; and e, *PyERF4.2*-RNAi. The concentration and volume of Agrobacterium solution used were the same for each infiltration site. Scale bars = 1 cm. H) The difference in color is indicated by the values of L*, a*, and b*. L* indicates luminance; a* indicates a range from green to magenta; b* indicates a range from yellow to blue. Bars indicate mean values ± SD from 6 biological replicates. I, J) Relative expression of *PyERF4.1* and *PyERF4.2*. Data are presented as means ± SD (*n* = 3). K) Total anthocyanin contents in transformed pear peels. Bars indicate mean values ± SD from 3 biological replicates. L–P) Relative expression of *PyERF3, PyMYB114*, *PyDFR*, *PyANS*, and *PyUFGT*. Data are presented as means ± SD (*n* = 3). Statistical analysis was carried out with one-way ANOVA and Student's *t*-test, and significance was marked with asterisks (*P < 0.05) or (**P < 0.01) or different letters (*P* < 0.05).

To verify the function of PyERF4.1 and PyERF4.2 in regulating anthocyanin biosynthesis, *PyERF4.1* and *PyERF4.2* were overexpressed in pear calli. The obtained transgenic pear calli and wild-type (WT) pear calli were treated with light, and then total anthocyanin contents were measured. After 15 d of light treatment, WT pear calli produced abundant anthocyanins, *PyERF4.1*-OE transgenic calli had no anthocyanin accumulation, while *PyERF4.2*-OE transgenic calli produced a little anthocyanin (Fig. 3A). The reverse transcription quantitative PCR (RT-qPCR) analysis showed that *PyERF4.1* and *PyERF4.2* were overexpressed in transgenic pear calli, respectively (Fig. 3, C and D). Compared to the WT calli, *PyERF4.1*-OE and *PyERF4.2*-OE calli showed significantly lower anthocyanin contents and a* values (*P* < 0.05) (Fig. 3, B and E). In *PyERF4.1*-OE and *PyERF4.2*-OE calli, *PyERF3*, *PyMYB114*, *PyDFR*, *PyANS*, and *PyUFGT* expression levels were significantly reduced compared to WT calli (*P* < 0.05), and their expression levels were more reduced in *PyERF4.1*-OE calli than in *PyERF4.2*-OE calli (Fig. 3F). In summary, PyERF4.1 and PyERF4.2 negatively regulated anthocyanin biosynthesis in pear calli, and the inhibitory effect of PyERF4.1 was stronger than that of PyERF4.2.

In order to further verify the inhibitory function of PyERF4.1/PyERF4.2 in anthocyanin biosynthesis of red-skinned pears, *PyERF4.1* and *PyERF4.2* were transiently silenced in ‘Zaosu’ pears. For avoiding silencing other genes with similar sequences, specific coding fragments from the 3′ region of the TFs were selected and inserted into the pSAK277 vector, resulting in 2 constructs: *PyERF4.1*-RNAi and *PyERF4.2*-RNAi. In Fig. 3G, there was no pigmentation observed in the empty vector pSAK277, *PyERF4.1* and *PyERF4.2* transformed pear peels, while obvious pigmentations were observed in *PyERF4.1*-RNAi and *PyERF4.2*-RNAi transformed pear peels. Compared with the control pear peels, *PyERF4.1* and *PyERF4.2* expression levels were significantly reduced (*P* < 0.05) (Fig. 3, I and J) and *PyERF3*, *PyMYB114*, *PyDFR*, *PyANS*, and *PyUFGT* expression levels were significantly increased (*P* < 0.05) in *PyERF4.1*-RNAi and *PyERF4.2*-RNAi transformed pear peels as shown by RT-qPCR analysis (Fig. 3, L–P). In addition, total anthocyanin contents and a* value were also significantly elevated when *PyERF4.1*-RNAi and *PyERF4.2*-RNAi were transformed (*P* < 0.05) (Fig. 3, H and K), which was consistent with the phenotype. Therefore, these results further validated the function of PyERF4.1 and PyERF4.2 in inhibiting anthocyanin biosynthesis in pears.

### PyERF4.1 negatively regulates anthocyanin biosynthesis in tomato fruits

Due to the stronger inhibitory effect of PyERF4.1 than PyERF4.2, PyERF4.1 was overexpressed in tomato fruits and T2 generation *PyERF4.1* overexpressing (*ERF4.1*-OE) transgenic tomato plants were obtained (Supplemental Fig. S2). However, for the difficulties associated with stable pear transformation, we screened the homologous gene *SlERF4.1* (C-terminus with a complete EAR motif) in tomato and obtained a T2 generation with the homologous mutation of tomato plants (*erf4.1*) by CRISPR/Cas9 (Supplemental Figs. S2 and S3). The above transgenic tomato lines were used to investigate the regulatory role of ERF4.1 on anthocyanin biosynthesis in tomato fruits.

**Figure 4.**
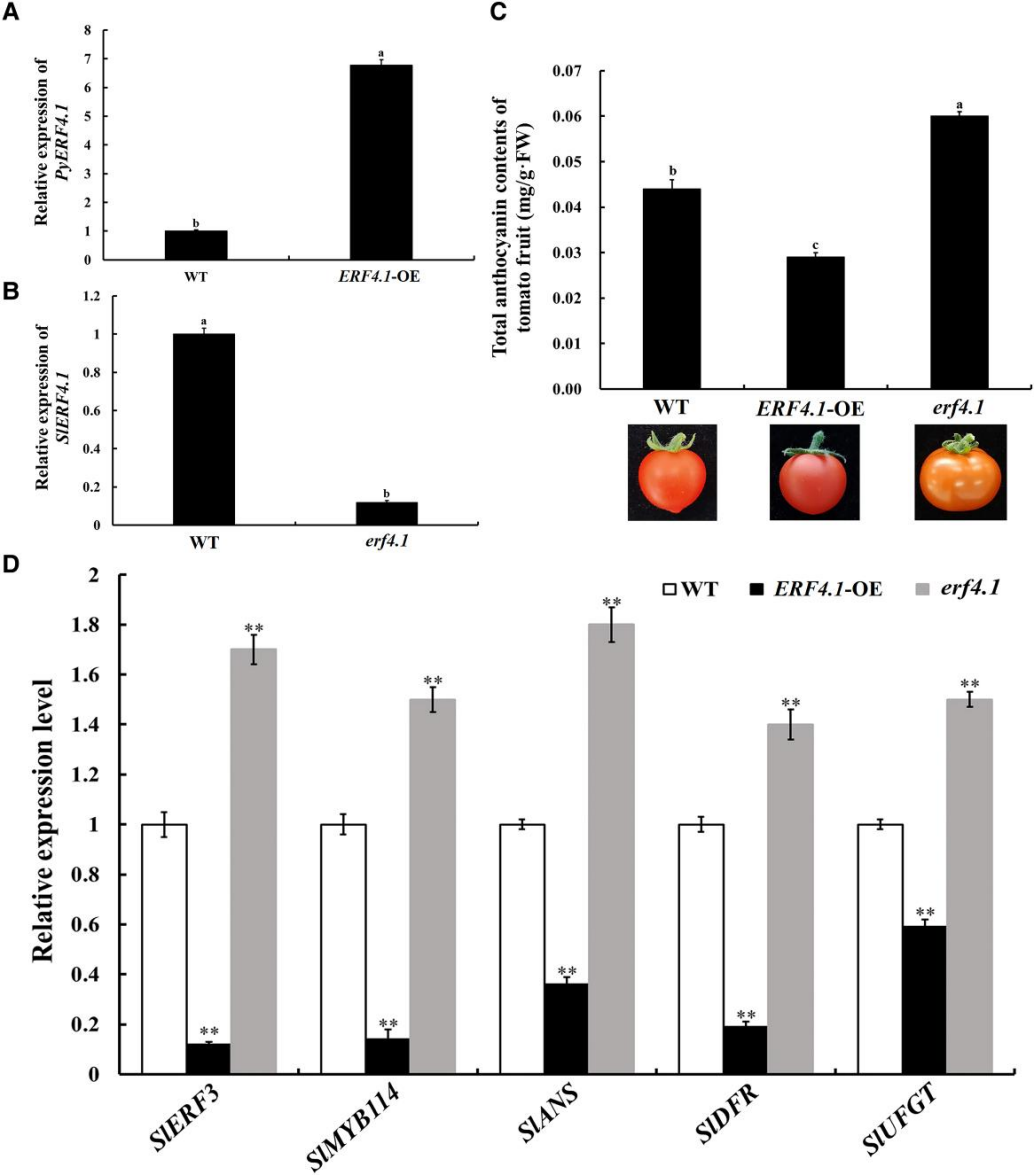
Validation of the inhibitory effect of PyERF4.1 on anthocyanin biosynthesis in transgenic tomato fruits. A, B) Relative expression of *PyERF4.1* and *SlERF4.1* in transgenic tomato fruits. WT, wild type; OE, overexpression; *erf4.1*, *ERF4.1* deletion mutant. Data are presented as means ± SD (*n* = 3). C) Total anthocyanin contents of *ERF4.1-*OE and *erf4.1* deletion mutant tomato fruits at red-ripening stage. Bars indicate mean values ± SD from 3 biological replicates. D) Relative expression of *SlERF3*, *SlMYB114*, *SlDFR, SlANS*, and *SlUFGT* in transgenic tomato fruits. Data are presented as means ± SD (*n* = 3). Statistical analysis was carried out with one-way ANOVA and Student's *t*-test, and significance was marked with asterisks (*P < 0.05) or (**P < 0.01) or different letters (*P* < 0.05).

RT-qPCR analysis revealed that *PyERF4.1* was significantly overexpressed in *ERF4.1*-OE tomato fruits (*P* < 0.05) (Fig. 4A), while *SlERF4.1* expression was significantly reduced in *erf4.1* tomato fruits (*P* < 0.05) (Fig. 4B), suggesting the success of genetic manipulation. Compared to that of WT fruits, the anthocyanins content of *ERF4.1*-OE fruits was significantly lower by 1.5 times (*P* < 0.05), whereas *erf4.1* fruits had a significantly higher anthocyanins content by 1.4 times (*P* < 0.05) (Fig. 4C). *SlERF3*, *SlMYB114* expression levels and anthocyanin biosynthesis-related genes *SlDFR*, *SlANS*, *SlUFGT* expression levels were significantly lower in *ERF4.1*-OE fruits than in WT fruits (*P* < 0.01), whereas they were significantly higher in *erf4.1* fruits than in WT fruits (*P* < 0.01) (Fig. 4D). In conclusion, ERF4.1 negatively regulated anthocyanin biosynthesis in tomato fruits.

### Co-transformation of PyERF4.1/PyERF4.2 with the PyERF3–PyMYB114–PybHLH3 complex substantially inhibits anthocyanin biosynthesis by transient expression system in pears

**Figure 5.**
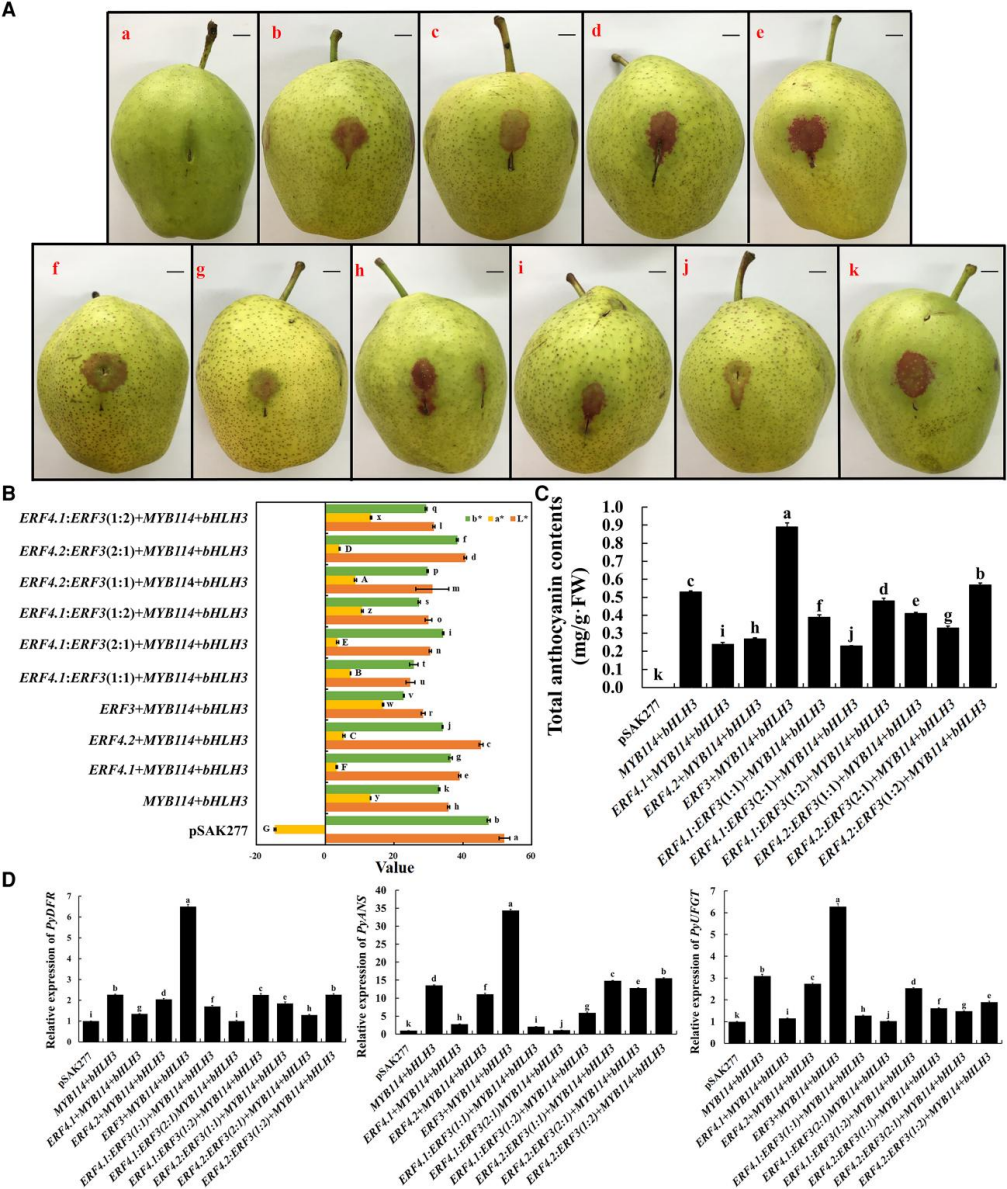
The inhibitory effect of *PyERF4.1*/*PyERF4.2* cotransformation with *PyERF3*, *PyMYB114*, and *PybHLH3* on anthocyanin biosynthesis by transient expression analysis in pear peels. A) The phenotype of ‘Zaosu’ pear peels after infiltration: a, pSAK277; b, *PyMYB114* + *PybHLH3*; c, *PyERF4.1* + *PyMYB114* + *PybHLH3;* d, *PyERF4.2* + *PyMYB114* + *PybHLH3*; e, *PyERF3* + *PyMYB114* + *PybHLH3*; f, *PyERF4.1*:*PyERF3* (1:1) + *PyMYB114* + *PybHLH3*; g, *PyERF4.1*:*PyERF3* (2:1) + *PyMYB114* + *PybHLH3*; h, *PyERF4.1*:*PyERF3* (1:2) + *PyMYB114* + *PybHLH3*; i, *PyERF4.2*:*PyERF3* (1:1) + *PyMYB114* + *PybHLH3*; j, *PyERF4.2*:*PyERF3* (2:1) + *PyMYB114* + *PybHLH3*; k, *PyERF4.2*:*PyERF3* (1:2) + *PyMYB114* + *PybHLH3*. Scale bars = 1 cm. B) The difference in color is indicated by the values of L*, a* and b*. L* indicates luminance; a* indicates a range from green to magenta; b* indicates a range from yellow to blue. Bars indicate mean values ± SD from 6 biological replicates. C) Total anthocyanin contents in transformed pear peels. Bars indicate mean values ± SD from 3 biological replicates. D) Relative expression of *PyDFR*, *PyANS*, and *PyUFGT*. Data are presented as means ± SD (*n* = 3). Statistical analysis was carried out with one-way ANOVA, and significance was marked with different letters (*P* < 0.05).

To investigate the inhibitory mechanism of PyERF4.1 and PyERF4.2 on anthocyanin biosynthesis in pears, a transient transformation of *PyERF4.1*/*PyERF4.2* and related TFs (*PyERF3*, *PyMYB114*, and *PybHLH3*) which are known as activators of the anthocyanin pathway, was performed in pear peels. Different ratios of *PyERF4.1*/*PyERF4.2*:*PyERF3* (1:1, 2:1, 1:2) and *PyMYB114–PybHLH3* were co-transformed into ‘Zaosu’ pear peels, and the phenotype was observed 6 d after injection. In Fig. 5A, there was no pigmentation seen in the empty vector pSAK277-transformed peels. Some pigmentation was observed when *PyMYB114–PybHLH3* were cotransformed, while substantial pigmentation was observed when *PyERF3–PyMYB114–PybHLH3* were cotransformed. Moreover, when the transformation ratio of *PyERF4.1*/*PyERF4.2* to *PyERF3* increased from 1:1 to 2:1, the pigment deposition gradually decreased, while when the transformation percentage of *PyERF3* increased, the pigment accumulation increased again. PyERF4.1 still had a stronger inhibitory effect on pigment accumulation than PyERF4.2. With the increase of *PyERF4.1*/*PyERF4.2* transformation, total anthocyanin contents and a* value decreased significantly (*P* < 0.05), which was consistent with the phenotypic changes after pear peels injection (Fig. 5, B and C).

Then, the major anthocyanin biosynthesis genes expression levels were analyzed in transfected pear peels by RT-qPCR. Consistent with the phenotypic results, PyERF4.1/PyERF4.2 significantly reduced *PyDFR*, *PyANS*, and *PyUFGT* expression levels after co-transformation with *PyERF3*–*PyMYB114*–*PybHLH3* (*P* < 0.05). Furthermore, this inhibitory effect enhanced as the amount of *PyERF4.1*/*PyERF4.2* transformation increased, while increasing the amount of *PyERF3* transformation could alleviate this inhibitory effect to some extent (Fig. 5D). Thus, *PyERF4.1/PyERF4.2* inhibits anthocyanin biosynthesis via potential interaction with *PyERF3*–*PyMYB114*–*PybHLH3* complex in pears.

### Heterologous expression of PyERF4.1/PyERF4.2 with a PyERF3–PyMYB114–PybHLH3 complex inhibits anthocyanin accumulation in strawberry receptacles

**Figure 6.**
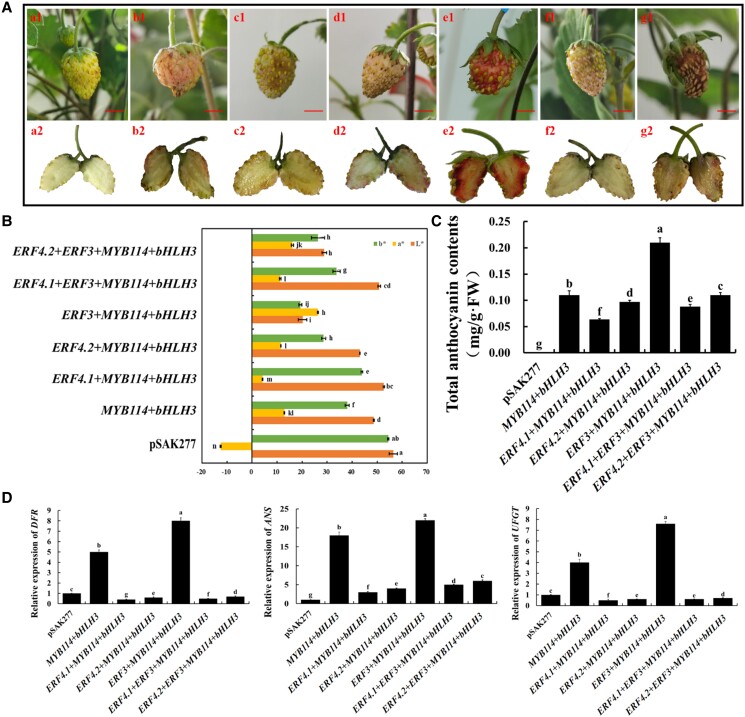
*PyERF4.1*/*PyERF4.2* cotransformation with *PyERF3*, *PyMYB114*, and *PybHLH3* inhibits anthocyanin biosynthesis by transient expression analysis in strawberry receptacles. A) The phenotype of strawberry receptacles after infiltration: a, pSAK277; b, *PyMYB114* + *PybHLH3*; c, *PyERF4.1* + *PyMYB114* + *PybHLH3;* d, *PyERF4.2* + *PyMYB114* + *PybHLH3*; e, *PyERF3* + *PyMYB114* + *PybHLH3*; f, *PyERF4.1* + *PyERF3* + *PyMYB114* + *PybHLH3*; g, *PyERF4.2* + *PyERF3* + *PyMYB114* + *PybHLH3*. 1, for the overall view. 2, for the section view. Scale bars = 5 mm. B) The difference in color is indicated by the values of L*, a*, and b*. L* indicates luminance; a* indicates a range from green to magenta; b* indicates a range from yellow to blue. Bars indicate mean values ± SD from 6 biological replicates. C) Total anthocyanin contents in transformed strawberry receptacles. Bars indicate mean values ± SD from 3 biological replicates. D) Relative expression of *FvDFR*, *FvANS*, *FvUFGT*. Data are presented as means ± SD (*n* = 3). Statistical analysis was carried out with one-way ANOVA, and significance was marked with different letters (*P* < 0.05).

For investigation of how PyERF4.1/PyERF4.2 regulates anthocyanin biosynthesis with PyERF3, PyMYB114, and PybHLH3, a transient transformation of their genes into strawberry receptacles was performed. The pigmentation appeared at the infiltration sites 6 d after injection, and the change trend was similar to that of pear peels. In Fig. 6A, there was no pigmentation observed in the empty vector pSAK277-transformed receptacles. Slight pigmentation was observed when *PyMYB114*–*PybHLH3* were cotransformed, while substantial pigmentation was observed when *PyERF3*–*PyMYB114*–*PybHLH3* were cotransformed. In contrast, when *PyERF4.1*/*PyERF4.2* were cotransformed with *PyMYB114*–*PybHLH3* or *PyERF3*–*PyMYB114*–*PybHLH3*, substantial inhibition of pigmentation was observed, and PyERF4.1 had a more pronounced inhibitory effect than PyERF4.2. In addition, differences in strawberry receptacles color affect L*, a*, and b* values (Fig. 6B). When *PyERF3*–*PyMYB114*–*PybHLH3* were cotransformed, anthocyanin contents in strawberry receptacles were significantly higher than when *PyMYB114*–*PybHLH3* were cotransformed (*P* < 0.05), while when *PyERF4.1*/*PyERF4.2* were cotransformed with *PyMYB114*–*PybHLH3* or *PyERF3*–*PyMYB114*–*PybHLH3*, the anthocyanin contents were significantly lower (*P* < 0.05) (Fig. 6C). Meanwhile, the anthocyanin content of strawberry receptacles cotransformed with *PyERF4.1* was always lower than that of strawberry receptacles cotransformed with *PyERF4.2* (*P* < 0.05). In conclusion, co-transformation of *PyERF4.1*/*PyERF4.2* with *PyERF3*–*PyMYB114*–*PybHLH3* substantially reduced anthocyanin biosynthesis in strawberry receptacles.

Next, RT-qPCR analysis showed that *PyERF4.1*/*PyERF4.2* significantly reduced anthocyanin biosynthesis-related genes (*FvDFR*, *FvANS*, and *FvUFGT*) expression levels in strawberry receptacles when cotransformed with *PyMYB114*–*PybHLH3* or *PyERF3*–*PyMYB114*–*PybHLH3* (*P* < 0.05) (Fig. 6D). Meanwhile, PyERF4.1 had a stronger inhibitory effect than PyERF4.2. Taken together, PyERF4.1/PyERF4.2 inhibits anthocyanin biosynthesis promoted by the PyERF3–PyMYB114–PybHLH3 complex in strawberry receptacles.

### PyERF4.1/PyERF4.2 interacts with PyERF3 through the EAR motif

**Figure 7.**
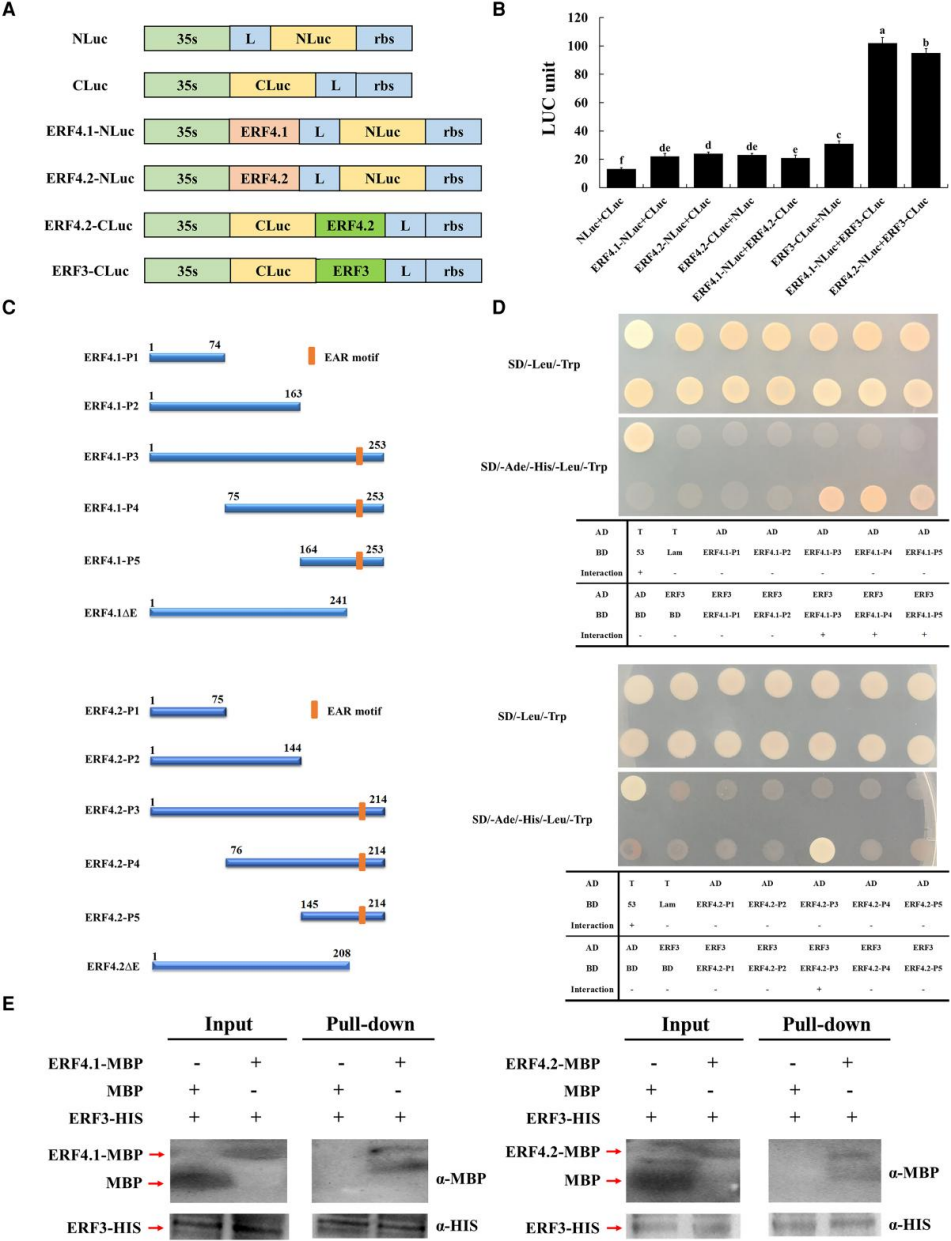

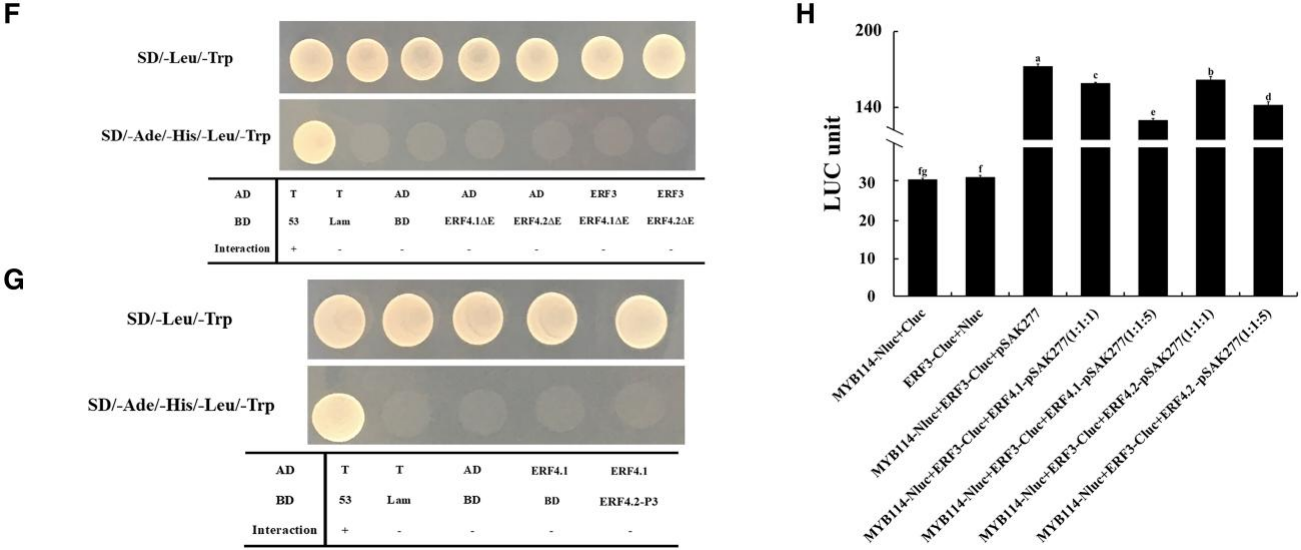
PyERF4.1/PyERF4.2 interacted with PyERF3 through the EAR motif. A) Model of the NLuc, CLuc, and NLuc/CLuc constructs. B) Verification of the interaction between PyERF4.1/PyERF4.2 and PyERF3 via FLC assays. Bars indicate mean values ± SD from 6 biological replicates. C) Protein fragment of PyERF4.1 and PyERF4.2. P1−P5, part1 to part5, the different amino acid residues of PyERF4.1 and PyERF4.2; EAR motif, ERF-associated-amphiphilic repression motif; ΔE, EAR motif deletion. D) Interaction of PyERF4.1/PyERF4.2 with PyERF3 verified by Y2H assays. E) Validation of PyERF4.1/PyERF4.2 and PyERF3 interactions with pull-down assays. *α*-MBP, MBP antibody; *α*-HIS, HIS antibody. F) Interaction of PyERF4.1/PyERF4.2 with PyERF3 through the EAR motif verified by Y2H assays. G) Interaction of PyERF4.1 with PyERF4.2 verified by Y2H assays. H) A luciferase complementation assay showing that PyERF4.1/PyERF4.2 weakened the interaction between PyERF3 and PyMYB114. Bars indicate mean values ± SD from 6 biological replicates. Statistical analysis was carried out with one-way ANOVA, and significance was marked with different letters (*P* < 0.05).

In order to explore the potential interaction between PyERF4.1/PyERF4.2 and PyERF3, PyMYB114, PybHLH3, a *Nicotiana benthamiana*-based firefly luciferase complementation (FLC) assay was performed. *PyERF4.1* and *PyERF4.2* were inserted into the NLuc, while *PyERF3*, *PyMYB114*, and *PybHLH3* were inserted into the CLuc, respectively (Fig. 7A; Supplemental Fig. S4A). Co-expression of PyERF4.1/PyERF4.2-NLuc and PyERF3-CLuc showed strong luciferase enzyme activity. On the contrary, no substantial luciferase enzyme activity was observed for other groups, including PyERF4.1/PyERF4.2-NLuc with CLuc, PyERF4.2/PyERF3-CLuc with NLuc, and PyERF4.1-NLuc with PyERF4.2-CLuc (Fig. 7B). When PyERF4.1/PyERF4.2-NLuc and PyMYB114-CLuc or PybHLH3-CLuc were co-expressed, no substantial luciferase enzyme activity was observed (Supplemental Fig. S4, B and C). Thus, it could be concluded that PyERF4.1/PyERF4.2 showed interactions with PyERF3 and no interactions with PyMYB114 and PybHLH3. Moreover, there was no interaction between PyERF4.1 and PyERF4.2.

Next, the interaction of PyERF4.1/PyERF4.2 with PyERF3, PyMYB114, and PybHLH3 was verified by yeast two-hybrid (Y2H) assays. *PyERF4.1* and *PyERF4.2* were inserted into pGBKT7 as full-length cDNA or as C-/N-terminal deletions (Fig. 7C), and *PyERF3*, *PyMYB114*, and *PybHLH3* full-length cDNAs were inserted into pGADT7. As shown in Fig. 7D, the 5 parts of PyERF4.1/PyERF4.2 (P1–P5) and PyERF3 did not show reporter activity in yeast. When the full-length PyERF4.1^1–253^ (P3) and 2 C-terminal parts PyERF4.1^75–253^ (P4), PyERF4.1^164–253^ (P5) were cotransformed with PyERF3, there was growth on both SD/-Trp/-Leu and SD/-Trp/-Leu/-His/-Ade medium. Thus, the interaction fragment of PyERF4.1 with PyERF3 was located within the C-terminal 90 amino acids (residues 164–253, P5). Meanwhile, when the full-length part PyERF4.2^1–214^ (P3) was cotransformed with PyERF3, there was growth on both SD/-Trp/-Leu and SD/-Trp/-Leu/-His/-Ade medium. Furthermore, none of the 5 parts of PyERF4.1 and PyERF4.2 showed reporter activity when cotransformed into yeast with PyMYB114 or PybHLH3 (Supplemental Fig. S4, D–G). Taken together, these results were consistent with the FLC assay, suggesting that PyERF4.1/PyERF4.2 have interactions with PyERF3 and no interactions with PyMYB114 and PybHLH3.

A pull-down assay was used to further validate the interaction of PyERF4.1/PyERF4.2 with PyERF3. *PyERF4.1* and *PyERF4.2* were constructed into the pMAL-c2x vector with MBP protein tag, and *PyERF3* was constructed into the pCold-TF vector with HIS protein tag. As shown in Fig. 7E, PyERF4.1 and PyERF4.2 proteins with MBP tag could be pulled down by PyERF3 protein with HIS tag, indicating that there is protein interaction of PyERF4.1/PyERF4.2 with PyERF3, which was consistent with the results of the FLC and Y2H assays.

Y2H assays showed that PyERF4.1 interacts with PyERF3 through the C-terminal 90 amino acids, the full-length of PyERF4.2 interacts with PyERF3, while the EAR motif of PyERF4.1 and PyERF4.2 were located at the C-terminus. Therefore, to verify whether PyERF4.1 and PyERF4.2 interact with PyERF3 through EAR motif, Y2H assay was performed on 2 fragments with deleted EAR motif, named as PyERF4.1ΔE and PyERF4.2ΔE (Fig. 7C). The interaction of PyERF4.1/PyERF4.2 with PyERF3 disappeared after the EAR motif deletion, indicating that PyERF4.1/PyERF4.2 interacts with PyERF3 through EAR motif. Besides, Y2H assay also demonstrated no interaction between PyERF4.1 and PyERF4.2 (Fig. 7, F and G).

To investigate the effect of PyERF4.1/PyERF4.2 on the interaction between PyMYB114 and PyERF3, a luciferase complementation assay was performed. The constructed PyMYB114-Nluc, PyERF3-Cluc. and PyERF4.1/PyERF4.2-pSAK277 recombinant vectors were coinfiltrated into *N. benthamiana* leaves. Co-expressed PyMYB114-Nluc and PyERF3-Cluc showed strong luciferase enzyme activity, but not in the negative controls. The luciferase enzyme activity significantly reduced when PyERF4.1/PyERF4.2-pSAK277 was added (*P* < 0.05), and the addition of PyERF4.1/PyERF4.2-pSAK277 was inversely associated with the luciferase enzyme activity (Fig. 7H). In summary, it could be concluded that the interaction between PyERF4.1/PyERF4.2 and PyERF3 interferes with the binding of PyERF3 to PyMYB114, which in turn affects the stability of PyERF3–PyMYB114–PybHLH3 complex.

### PyMYB114 activates the PyERF4.1/PyERF4.2 transcription and regulates the expression of anthocyanin biosynthesis genes in pears

**Figure 8.**
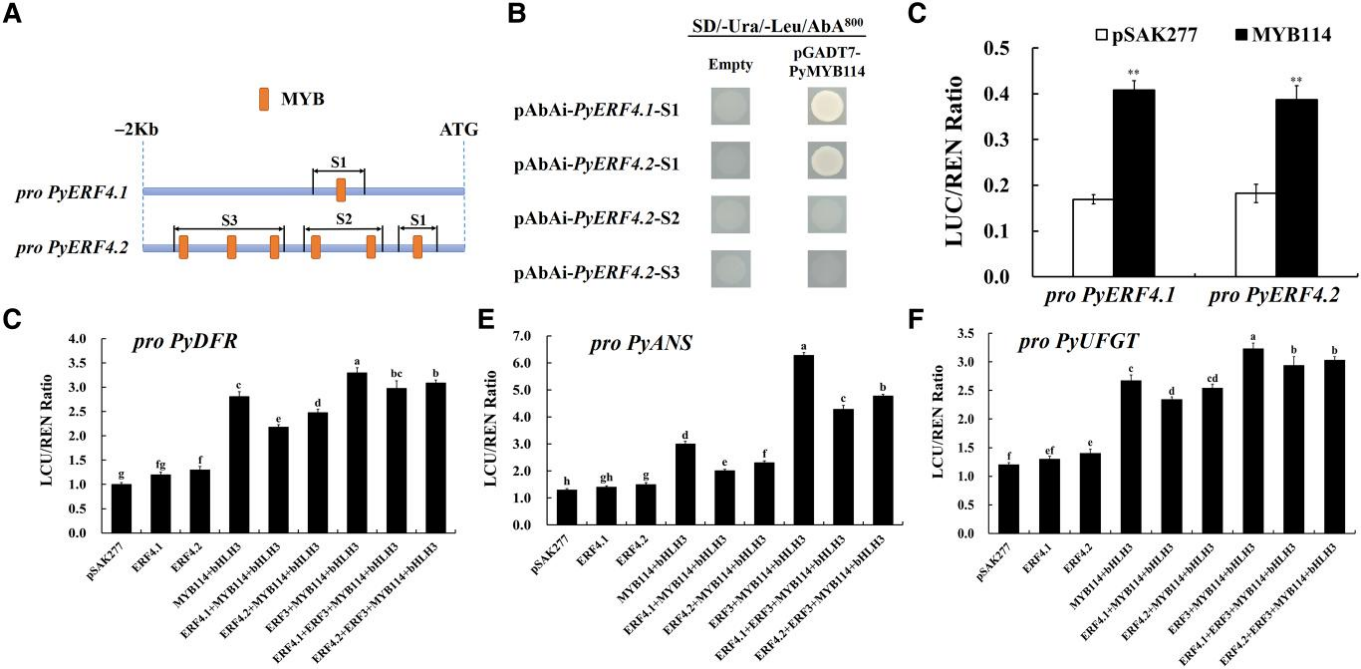
PyMYB114 binds to the *PyERF4.1*/*PyERF4.2* promoter and regulates transcription of anthocyanin biosynthesis genes. A) Schematic of the *PyERF4.1* and *PyERF4.2* promoters. The cis-acting elements of the promoter region were predicted using the PlantPAN 3.0 database and segmented (S1−S3). Pro, promoter. The MYB is MYBs cis-acting element. B) Y1H assays between PyMYB114 and the *PyERF4.1*/*PyERF4.2* promoter. The yeast colonies were screened on SD/−Ura/−Leu/AbA^800^ plates. C) The dual-luciferase reporter assay verifies PyMYB114 binding to *PyERF4.1* and *PyERF4.2* promoters. Data are presented as means ± SD (*n* = 6). D–F) PyERF4.1/PyERF4.2 cotransformation with PyERF3, PyMYB114 and PybHLH3 on the transcription of *PyDFR*, *PyANS*, and *PyUFGT* verified by the dual-luciferase reporter assay. Data are presented as means ± SD (*n* = 6). Statistical analysis was carried out with one-way ANOVA and Student's *t*-test, and significance was marked with asterisks (*P < 0.05) or (**P < 0.01) or different letters (*P* < 0.05).

Based on the results of transient expression systems in pear peels and strawberry receptacles, it was found that *PyERF4.1*/*PyERF4.2* cotransformation with *PyMYB114*–*PybHLH3* inhibited pigment deposition, but neither PyERF4.1 nor PyERF4.2 interacted with PyMYB114 and PybHLH3. Furthermore, *PyERF4.1* and *PyERF4.2* expression levels were significantly elevated when *PyMYB114* and *PybHLH3* were transiently overexpressed (*P* < 0.01) (Supplemental Fig. S5A), and PybHLH3 could not activate the *PyERF4.1* and *PyERF4.2* transcription (Supplemental Fig. S5B). Therefore, it was hypothesized that PyMYB114 binds to the *PyERF4.1* and *PyERF4.2* promoters, and this regulatory module was verified with yeast one-hybrid (Y1H) assay. Cis-acting elements on the promoter sequence of *PyERF4.1* and *PyERF4.2* were predicted by PlantPAN 3.0, and the results are presented in Fig. 8A. The upstream 2 kb promoter region of the *PyERF4.1* and *PyERF4.2* was found to contain one or more cis-acting elements of MYB TFs. Then, the promoter segment baits were integrated with the prey vectors pGADT7-PyMYB114 and introduced into the Y1HGold yeast strain, and the results showed that PyMYB114 could bind to the S1 fragment of *PyERF4.1*/*PyERF4.2* (Fig. 8B). The trans-activity of PyMYB114 to the *PyERF4.1*/*PyERF4.2* was further verified by a dual-luciferase reporter assay in *N. benthamiana* leaves. In Fig. 8C, the transformation of *PyMYB114* had a significant activation effect on the transcription of *PyERF4.1* and *PyERF4.2* compared to that of the empty vector pSAK277 (*P* < 0.01). In conclusion, these results indicated that PyMYB114 binds to the *PyERF4.1* and *PyERF4.2* promoters, thereby activating their transcription.

PyERF4.1/PyERF4.2 interacted with PyERF3, PyMYB114, and PybHLH3 to inhibit anthocyanin biosynthesis, but the downstream target genes on which they act remain to be determined. Therefore, the dual-luciferase reporter assay in *N. benthamiana* leaves was performed to investigate the effect of *PyERF4.1*/*PyERF4.2* cotransformation with *PyERF3*, *PyMYB114*, and *PybHLH3* on the downstream target genes *PyDFR*, *PyANS*, and *PyUFGT*, which are associated with anthocyanin biosynthesis. In contrast to the empty vector pSAK277 transformation, *PyERF3*, *PyMYB114*, and *PybHLH3* cotransformation was found to significantly activate *PyDFR*, *PyANS*, and *PyUFGT* promoters, whereas the activation was significantly attenuated when cotransformed with *PyERF4.1*/*PyERF4.2* (*P* < 0.05) (Fig. 8, D–F). Also, the de-activating effect of PyERF4.1 on *PyANS* promoters was significantly stronger than that of PyERF4.2 (*P* < 0.05). Therefore, PyERF4.1/PyERF4.2 damaged the activating effect of the complex PyERF3–PyMYB114–PybHLH3 on the promoters of anthocyanin biosynthesis genes.

### EAR motif deletion or mutation diminishes the inhibitory effect of PyERF4.1 and PyERF4.2 on anthocyanin biosynthesis in red-skinned pears

**Figure 9.**
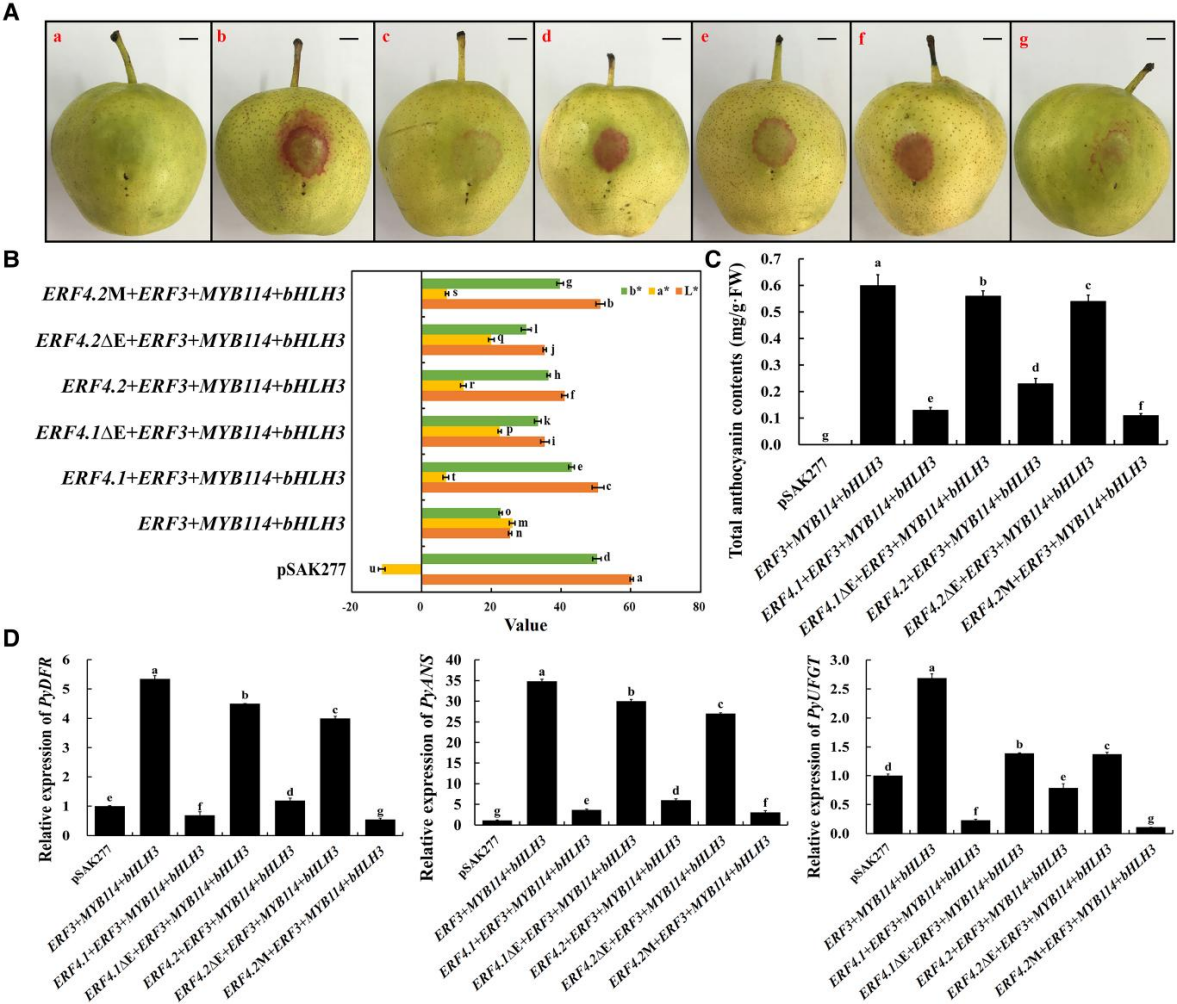
Effect of EAR motif integrity on the function of PyERF4.1 and PyERF4.2 in inhibiting anthocyanin biosynthesis. A) The phenotype of ‘Zaosu’ pear peels after infiltration: a, pSAK277; b, *PyERF3* + *PyMYB114* + *PybHLH3*; c, *PyERF4.1* + *PyERF3* + *PyMYB114* + *PybHLH3;* d, *PyERF4.1*ΔE + *PyERF3* + *PyMYB114* + *PybHLH3*; e, *PyERF4.2* + *PyERF3* + *PyMYB114* + *PybHLH3*; f, *PyERF4.2*ΔE + *PyERF3* + *PyMYB114* + *PybHLH3*; g, *PyERF4.2* M + *PyERF3* + *PyMYB114* + *PybHLH3*. ΔE, EAR motif deletion. M, EAR motif mutation. Scale bars = 1 cm. B) The difference in color is indicated by the values of L*, a*, and b*. L* indicates luminance; a* indicates a range from green to magenta; b* indicates a range from yellow to blue. Bars indicate mean values ± SD from 6 biological replicates. C) Total anthocyanin contents in transformed pear peels. Bars indicate mean values ± SD from 3 biological replicates. D) Relative expression of *PyDFR*, *PyANS*, and *PyUFGT*. Data are presented as means ± SD (*n* = 3). Statistical analysis was carried out with one-way ANOVA, and significance was marked with different letters (*P* < 0.05).

Although PyERF4.1 and PyERF4.2 could inhibit anthocyanin biosynthesis in pears, PyERF4.1 showed stronger inhibition, presumably because it had a complete EAR motif, whereas PyERF4.2 had an amino acid mutation in its EAR motif. To verify this hypothesis, the EAR motifs of PyERF4.1/PyERF4.2 were deleted (*PyERF4.1*ΔE/*PyERF4.2*ΔE), and the Met residue was mutated to the Leu residue in the EAR motif of PyERF4.2 (*PyERF4.2*M) to mimic an intact EAR motif, and the vectors were cotransformed with *PyERF3*, *PyMYB114*, and *PybHLH3* into ‘Zaosu’ pears (Supplemental Fig. S6). When *PyERF4.1*ΔE or *PyERF4.2*ΔE were cotransformed with *PyERF3*–*PyMYB114*–*PybHLH3*, the same substantial pigment deposition occurred as when *PyERF3*–*PyMYB114*–*PybHLH3* were cotransformed. In contrast, when *PyERF4.2*M was cotransformed with *PyERF3*–*PyMYB114*–*PybHLH3*, less pigmentation appeared than when cotransformed with *PyERF4.2* (Fig. 9A). The color changes and anthocyanin contents of the injection sites were consistent with the phenotypes (Fig. 9, B and C). RT-qPCR analysis showed that the *PyDFR*, *PyANS*, and *PyUFGT* expression level were significantly higher when *PyERF3*–*PyMYB114*–*PybHLH3* were cotransformed or when *PyERF4.1*ΔE/*PyERF4.2*ΔE was cotransformed (*P* < 0.05), while when *PyERF3*–*PyMYB114*–*PybHLH3* were cotransformed with *PyERF4.1*/*PyERF4.2* or *PyERF4.2*M, they were significantly reduced (*P* < 0.05) (Fig. 9D). In summary, it could be concluded that EAR motif deletion diminishes the inhibitory effect of PyERF4.1 and PyERF4.2 on anthocyanin biosynthesis, whereas a mutation of the EAR motif in PyERF4.2 to intact EAR motif (LxLxM to LxLxL) strengthened the inhibitory effect on anthocyanin biosynthesis in red-skinned pears.

## Discussion

### PyERF4.1 and PyERF4.2 inhibit anthocyanin biosynthesis in red-skinned pears

Recently, red-skinned pear is popular because of rich anthocyanins, but its coloring is unstable during the development process, thus the molecular mechanism of anthocyanin biosynthesis in many species has been widely concerned and studied (Li et al. 2020). Many AP2/ERF TFs have been reported to positively regulate anthocyanin biosynthesis in plants, such as AtERF4 and AtERF8 in Arabidopsis (*Arabidopsis thaliana*) (Koyama and Sato 2018), MdERF1B (Zhang et al. 2018), MdERF3 (An et al. 2018), and MdERF38 (An et al. 2020b) in apple. In red-skinned pear, PyERF3 (Yao et al. 2017), Py4ERF24, Py12ERF96 (Ni et al. 2019), PyERF22 (Wu et al. 2020), and PyERF105 (Ni et al. 2021) all promote anthocyanin biosynthesis. The negative regulation of pigment metabolism by ERFs is equally important, but little attention has been paid to it. Most of the available reports are about the regulation of the color change in fruit ripening process, such as tomato SlERF6 and SlERF.F12 (Lee et al. 2012; Deng et al. 2022), loquat (*Eriobotrya japonica*) EjERF11 (Zeng et al. 2015), banana (*Musa nana* Lour.) MaERF11 (Han et al. 2016), and apple (*Malus domestica*) MdERF4 (Hu et al. 2022).

In this study, expression of 2 *AP2/ERFs*, *PyERF4.1* and *PyERF4.2* screened from the transcriptome data of ‘Starkrimson’ pear and its green mutant during 3 fruit developmental periods was substantially and negatively correlated with anthocyanin contents as well as *PyERF3*, *PyMYB114*, *PybHLH3*, *PyDFR*, *PyANS*, and *PyUFGT* expression by bioinformatics and correlation analysis (Figs. 1 and 2). PyERF4.1 and PyERF4.2 as repressors regulated anthocyanin biosynthesis in overexpression pear calli and stable hereditary tomato fruits (Figs. 3 and 4). Further studies indicated that PyERF4.1 and PyERF4.2 inhibited anthocyanin biosynthesis with PyERF3–PyMYB114–PybHLH3 complex in strawberry receptacles and pears as verified by transient expression and RT-qPCR analysis (Figs. 5 and 6). Pigment deposition is a phenotypic feature during fruit ripening. The present study verified that PyERF4.1 and PyERF4.2 inhibited anthocyanin biosynthesis in red-skinned pear fruit, which provides a more in-depth study for the molecular mechanism of ERFs negatively regulating the color change in fruit-ripening process. In summary, we provided direct evidence highlighting the involvement of PyERF4.1 and PyERF4.2 as repressors of anthocyanin biosynthesis in red-skinned pears.

### The inhibitory effect of AP2/ERFs on anthocyanin biosynthesis is dependent on EAR motif in pears

EAR motif-mediated repression of transcription is the predominant form of repression in plants (Kagale et al. 2010; Choi et al. 2018), and an increasing amount of studies have reported that deletion and mutation of EAR motif affect the function of genes, e.g. a single amino acid mutant in the EAR motif of IbMYB44.2 reduced the inhibition of anthocyanin accumulation in the purple-fleshed sweet potato (*Ipomoea batatas* L.) (Li et al. 2021); EAR motif mutation (C-G) in MdERF4 reduces the expression of *MdERF3*, and promoted apple fruit ripening (Hu et al. 2022). In this study, the PyERF4.1 amino acid sequence had an intact EAR motif at the C-terminus (DLNLxP), while the PyERF4.2 amino acid sequence had an incomplete EAR motif with a mutated amino acid (LxLxM) (Fig. 1C). Moreover, PyERF4.1 was verified to have a stronger inhibitory effect than PyERF4.2 in strawberry receptacles, pear fruits, and transgenic pear calli (Figs. 3, 5, and 6), presumably due to the incomplete EAR motif of PyERF4.2. This is similar to previous studies reporting that a single amino acid mutation in the EAR motif diminishes the inhibitory effect. Some studies reported that the deletion or mutation of the EAR motif abolished the cell death-inducing ability of StERF3 in potato (*Solanum tuberosum*) and the inhibitory effect of SlERF.F12 on ripening in tomato (Deng et al. 2022; Qi et al. 2022). Here, transient expression assays in pears of *PyERF4.1* and *PyERF4.2* with EAR motif deletions showed that EAR motif deletion abolished the inhibitory effect of PyERF4.1/PyERF4.2 on anthocyanin biosynthesis, whereas the inhibitory effect of PyERF4.2 was enhanced after incomplete EAR motif mutated to an intact form (Fig. 9). These results demonstrate that EAR motif deletion diminishes the inhibitory effect of PyERF4.1/PyERF4.2 on anthocyanin biosynthesis in pears. Consistently, previous studies reported that EAR motif deletion or mutation promotes ripening in tomato and apple by Deng et al. (2022) and Hu et al. (2022).

### A regulatory model of a feedback regulatory loop balancing anthocyanin biosynthesis in red-skinned pears

The MBW complex is a major regulator in the plant anthocyanin biosynthetic pathway and has been widely reported in tomato (Yan et al. 2020), apple (An et al. 2020a), and pear (Cui et al. 2021). An increasing number of studies have reported that AP2/ERFs regulate anthocyanin biosynthesis in pears through the MBW complex (Ni et al. 2019, 2021; Wu et al. 2020). This study verified that PyMYB114 binding to the promoter of *PyERF4.1*/*PyERF4.2*, activating their transcription and leading to a decrease in transcriptional activity of anthocyanin biosynthesis key gene *PyANS*, thereby inhibiting red-skinned pear fruit coloration (Fig. 8). Previous studies reported that MdMYB1 activates the promoter of transcriptional activator *MdERF3* to promote ethylene production in apples (An et al. 2018). In the present study, PyMYB114 activates the promoter of transcriptional repressor *PyERF4.1*/*PyERF4.2* to inhibit anthocyanin biosynthesis in red-skinned pears. This is similar to previous studies, while MYBs activate the promoter of repressor which provides a distinct module for the mechanism of red-skinned pear coloration regulation.

**Figure 10.**
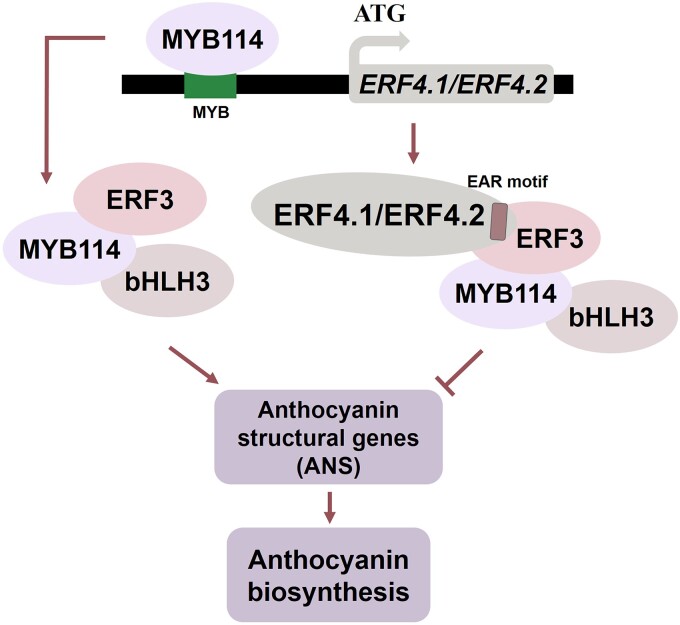
A regulatory model for PyERF4.1 and PyERF4.2 inhibition of anthocyanin biosynthesis in red-skinned pears.

It has been shown that PyERF3 interacts with PyMYB114 and forms the PyERF3–PyMYB114–PybHLH3 complex to activate downstream target genes and enhance anthocyanin biosynthesis in red-skinned pears (Yao et al. 2017). In this study, PyERF4.1 and PyERF4.2 were found to interact with PyERF3 through EAR motif to affect the stability of the PyERF3–PyMYB114–PybHLH3 complex and thus inhibit red-skinned pears anthocyanin biosynthesis (Fig. 7). This model of regulation has similarities with the interaction of MdERF2 and MdERF3 proteins that inhibits the association of MdERF3 to the *MdACS1* promoter, thereby negatively affecting ethylene biosynthesis and fruit ripening in apples, as previously reported by Li et al. (2016). Furthermore, we found that PyMYB114 could not only promote anthocyanin accumulation via forming a complex with PybHLH3–PyERF3, but also activate the repressors *PyERF4.1* and *PyERF4.2* to affect the stability of PyERF3–PyMYB114–PybHLH3 complex, leading to a decrease in the accumulation of anthocyanin in pears. Thus, a feedback regulation loop was formed to balance the excessive accumulation of anthocyanins in red-skinned pears. In summary, a regulatory model of *PyERF4.1*/*PyERF4.2* inhibition of anthocyanin biosynthesis in red-skinned pears can be summarized: PyMYB114 binds to the promoter of *PyERF4.1*/*PyERF4.2* and activates its transcription in order to balance the accumulation of anthocyanin in red-skinned pears, then PyERF4.1/PyERF4.2 interact with PyERF3 through the EAR motif to break the stability of the PyERF3–PyMYB114–PybHLH3 complex, which inhibited anthocyanin biosynthesis gene *PyANS* transcription, resulting in the decreased anthocyanin in red-skinned pears (Fig. 10).

## Materials and methods

### Plant materials and growth conditions

The red-skinned pears ‘Hongzaosu’ (*P. pyrifolia* Nakai) and the green-skinned pears ‘Zaosu’ (*P. pyrifolia* Nakai) used in this study were provided by Shandong Institute of Pomology (Tai’an, China). The fruits were harvested at 30, 60, 90 DAFB, photographed and sampled for peels, frozen in liquid nitrogen and stored at −80 °C. The ‘Zaosu’ pears at ripening stage and diploid strawberry (*F. vesca*) ‘Yellow Wonder’ 5AF7 at 2 weeks after flowering grown in a greenhouse under artificial light (16 h at 25 °C in the day and 8 h at 18 °C in the night), were used for transient expression assays. The 4-week-old tobacco (*N. benthamiana*) leaves were used for dual-luciferase reporter system and FLC assays. The phenotype of pear peels, tobacco leaves, and strawberry receptacles were observed 6 d postinjection, and then the samples were frozen in liquid nitrogen and stored at −80 °C.

Tomato (*S. lycopersicum*) *cv*. Micro-Tom was selected as the WT in this study. The WT, overexpression and deletion mutation tomato plants were grown in a greenhouse under artificial light (16 h at 25 °C in the day and 8 h at 18 °C in the night). Red ripening tomato fruits of stable lines with T2 generation overexpression (*ERF4.1*-OE) and homozygous mutants (*erf4.1*) were sampled and frozen in liquid nitrogen and stored at −80 °C. Each line sample contained at least 6 fruits.

### Bioinformatic analysis

The 114 differentially expressed genes AP2/ERFs from transcriptome data of red-skinned pear ‘Starkrimson’ (*P. communis* L.) and its green mutant reported by Yang et al. (2015) were analyzed. Heat map was established with the R script, and the phylogenetic tree was constructed by the MEGA7 Program according to the neighbor-joining method, bootstrap analysis (1,000 replicates). The amino acid sequence alignment analysis was performed through the DNAMAN Program.

### RT-qPCR analysis

Total RNA was extracted from pear peel, strawberry receptacle, pear calli, and tomato fruit samples by the Plant RNA Isolation Kit (Foregene, Chengdu, China), and first-strand RNA was synthesized using a Primer Reverse Transcription Master Mix Kit (Takara, Tokyo, Japan). Primers for RT-qPCR are listed in Supplemental Table S1. *PyTubulin*, *PyActin*, *Fv26S*, *FvActin*, *SlTubulin*, and *SlActin* expression levels were used as normalized genes, and relative genes expression was determined by the 2^−ΔΔ*CT*^ method (Pirrello et al. 2006). Three biological replicates were used for all analyses.

### Genes cloning and recombinant vectors construction

Full-length sequences of *PyERF4.1* and *PyERF4.2* genes cloned from the cDNA of ‘Starkrimson’ pears. For the construction of stable genetic lines, full-length coding sequences of *PyERF4.1* and *PyERF4.2* were inserted into the pCAMBIA1300-221 vector at the restriction sites of *Bam*HI and *Sac* I under the control of 35S promoter. For the transient transformed expression analysis, full-length coding sequences of *PyERF4.1*, *PyERF4.2*, *PyERF3*, *PyMYB114*, and *PybHLH3*, and the sequences of *PyERF4.1*ΔE (bases 1–723 bp) and *PyERF4.2*ΔE (bases 1–624 bp) with deleted EAR motif as well as *PyERF4.2*M (methionine mutated to leucine) with mutated EAR motif were inserted into the pSAK277 vector at the restriction sites of *Eco*R I and *Xba* I under the control of 35S promoter. For RNA interference (RNAi)-induced gene expression silencing, a 300 bp *PyERF4.1*-specific DNA fragment (bases 374–673 bp) and a 279 bp *PyERF4.2*-specific DNA fragment (bases 364–642 bp) were inserted into the pSAK277 vector, respectively. Primers used to construct the recombinant plasmids are listed in Supplemental Table S2.

### Transient transformation and RNAi-induced silencing in pear fruits and heterologous expression in strawberry receptacles

For the transient transformed expression and RNAi-induced silencing analysis, the recombinant plasmids constructed on the pSAK277 vector were transformed into GV3101 strains of *Agrobacterium tumefaciens*, and then Agrobacterium cells containing recombinant pSAK277 vector were resuspended in the injection solution containing 10 mmol 2-(*N*-morpholino)ethanesulfonic acid hydrate (MES), 10 mmol MgCl_2_ and 0.2 µmol acetosyringone (OD_600_ = 1.0), then cultured at 25 °C under 70 rpm for 4 h before injection. The empty vector (pSAK277) was used as a negative control. The detail of the infiltration experiment was carried out following the description by Yao et al. (2017). Strawberry receptacles and pear fruits were incubated in the dark for 24 h after injection, then moved to artificial light (16 h of daylight), and incubated at 24 °C. After 7 d, pear peels and strawberry receptacles were collected to determine anthocyanin content and for total RNA extraction.

### Generation of transgenic pear calli

The induced calli from the flesh of ‘Clapp's Favorite’ (*P. communis* L.) were subcultured several times, and the rapidly growing soft pear calli was selected and played under dark condition on the Murashige and Skoog (MS) solid medium supplemented with 30 g/L sucrose, 0.5 mg/L 6-benzylaminopurine, and 1.0 mg/L 2,4-dichlorophenoxyacetic acid. Pear calli was transformed as previously reported (Bai et al. 2019). Pear calli were immersed in *A. tumefaciens* strain EHA105 containing pCAMBIA1300-221-*PyERF4.1* or pCAMBIA1300-221-*PyERF4.2* recombinant plasmids for 30 min with the empty vector pCAMBIA1300-221 as a negative control (WT). After 2 d of co-culture, the pear calli were cultured in darkness on the MS solid medium containing appropriate antibiotics for 1 mo at 24 °C and subcultured every 2 weeks. For the light treatment, fresh subcultured calli were treated with continuous light and observed after 7 d.

### Generation of stable overexpressed and deletion mutant tomato fruits

The constructed pCAMBIA1300-221-*PyERF4.1* recombinant plasmids was transformed into Micro-Tom via *A. tumefaciens* EHA105-mediated transformation. Stable lines of T2 generation of overexpression (*ERF4.1*-OE) was used for further analysis.

Due to the difficulties of stable pear transformation, homologous genes in tomato were searched using the BLAST program in NCBI with the coding sequence of *PyERF4.1* as a query (https://www.ncbi.nlm.nih.gov/). It was found that *SlERF4.1* (Solyc10g009110.1.1) showed high similarity to *PyERF4.1* (41.11% identity at the amino acid level). The pYLCRISPR/Cas9-DN binary vector for plant CRISPR/Cas9-mediated genome editing was gifted by Prof. Yaoguang Liu (South China Agricultural University). Two target sequences of *SlERF4.1* were designed using the CRISPR direct online tool (http://crispr.dbcls.jp/). The double-stranded DNA of target sequences was amplified by PCR and cloned into the pYLCRISPR/Cas9-DN binary vector using the Golden Gate ligation method (Engler and Marillonnet 2013). Primers used to construct the recombinant plasmids are listed in Supplemental Table S2.

The constructed pYLCRISPR/Cas9-DN-*SlERF4.1* recombinant plasmids were transformed into Micro-Tom via *A. tumefaciens* EHA105-mediated transformation. Stable lines of T2 generation of homozygous mutants (*erf4.1*) were used for further analysis.

### Extraction and determination of anthocyanin contents

The differences in color due to anthocyanins were indicated by L*, a*, and b* values determined by the colorimeter (WSC-100, Konica Minolta, Tokyo, Japan). The method described by Lee and Wicker (1991) was used to extract and determine the anthocyanin content. Briefly, 0.2 g of sample (pear peel, strawberry receptacle, pear calli, or tomato fruit) was homogenized with 1 mL of 1% (v/v) hydrochloric acid methanol solution. The absorbance was measured at 530, 620 and 650 nm by the Multiskan Spectrum (Thermo Scientific Multiskan GO 1510, Finland), and used the formula OD = (*A*_530_ − *A*_620_) − 0.1×(*A*_650_ − *A*_620_) to calculate total anthocyanin contents of each sample and expressed as mg/g fresh weight.

### Dual-luciferase reporter assay

For the construction of dual-luciferase reporter vector, the 2 kb promoter regions (from the ATG start codon) upstream of *PyERF4.1*, *PyERF4.2*, *PyDFR*, *PyANS*, and *PyUFGT* were cloned with the primers listed in Supplemental Table S2 and inserted into the pGreen II 0800-LUC binary vector. Furthermore, Agrobacterium transformation and injection were performed in the same way as for the pear fruit and strawberry transient transformation assays. Agrobacterium cells containing the pGreen II 0800-LUC and the pSAK277 recombinant plasmids were mixed in a 1:9 ratio. The Agrobacterium cells mixture was injected into the 4-week-old *N. benthamiana* leaves for transient transformation expression assay. The ratio of firefly luciferase (LUC)/*Renilla* luciferase (Ren) was determined by the Dual-Luciferase Reporter Assay System (E1910, Promega, USA).

### Firefly luciferase complementation assay

Firefly luciferase complementation (FLC) assay was performed as reported by Chen et al. (2008). Full-length coding sequences of *PyERF4.1* and *PyERF4.2* without the stop codons were cloned into the pCAMBIA1300-NLuc binary vector, and full-length sequences of *PyERF3* and *PyMYB114* were inserted into the pCAMBIA1300-CLuc binary vector. The primer sequences are listed in Supplemental Table S2. Agrobacterium transformation and injection were performed as described previously for transient transformation assays in *N. benthamiana* leaves. The firefly luciferase activity was measured after 72 h using the Steady-Glo Luciferase Assay System (E2510, Promega, USA).

### Y1H assay

Y1H assay was carried out with the Matchmaker Gold Yeast One-Hybrid System (630491, Clontech, Japan). One segment of the *PyERF4.1* promoter (−905 bp to −558 bp) and 3 segments of the *PyERF4.2* promoter (−2,000 bp to −1,887 bp, −1,640 bp to −1,281 bp, and −1,632 bp to −1,087 bp) were cloned into the pAbAi vector containing *Hind* III and *Xho* I, and the full sequence of *PyMYB114* was cloned into the pGADT7 vector containing *Eco*R I and *Xho* I. The primer sequences used for vector construction are listed in Supplemental Table S2. Then, the prey vectors were transformed into Y1H Gold cells containing the pAbAi-bait and detected on SD/-Ura/-Leu/AbA plates.

### Y2H assay

Y2H assay was carried out with the Matchmaker Gold Yeast Two-Hybrid System. Six fragments of *PyERF4.1* gene (ERF4.1^1–74^, ERF4.1^1–163^, ERF4.1^1–253^, ERF4.1^75–253^, ERF4.1^164–253^, and ERF4.1^1–241^), and 6 fragments of *PyERF4.2* gene (ERF4.2^1–75^, ERF4.2^1–144^, ERF4.2^1–214^, ERF4.2^76–214^, ERF4.2^145–214^, and ERF4.2^1–208^) were inserted into the pGBKT7 vector containing *Nde* I and *Pst* I, and the full sequences of *PyERF4.1*, *PyERF3*, and *PyMYB114* were cloned into pGADT7 at the restriction sites of *Eco*R I and *Xho* I. The primer sequences used for vector construction are listed in Supplemental Table S2. Then, the recombinant plasmids were cotransformed into Y2H Gold cells by LiCl-PEG method, and the interactions were detected on SD/-Leu/-Trp/-His/-Ade plates. The pGADT7-T and pGBKT7-53 or pGADT7-T and pGBKT7-Lam were cotransformed as positive and negative controls, respectively.

### Pull-down assay

Full-length coding sequences of *PyERF4.1* and *PyERF4.2* were cloned into pMAL-p5x and pMAL-c2x vectors with *Bam*H I and *Hind* III, and the full-length coding sequence of *PyERF3* was cloned into pCold TF DNA vector with *Bam*H I and *Xba* I. The primer sequences used for vector construction are shown in Supplemental Table S2. Next, PyERF4.1-MBP, PyERF4.2-MBP, and PyERF3-HIS fusion proteins were expressed in DE3 *Escherichia coli* cells, and PyERF3*-*HIS was purified using Ni Sepharose 6 Fast Flow (17-5318-06, GE Healthcare, Sweden). The collected elution extract was blotted with MBP or HIS antibodies (Beyotime, Shanghai, China).

### Accession numbers

Sequence data for this article is available in the genome database for the Rosaceae (http://www.rosaceae.org), the National Center for Biotechnology Information (NCBI, https://www.ncbi.nlm.nih.gov/), or the Solanaceae (https://solgenomics.net/) under the following accession numbers: *PyERF4.1* (ON652752), *PyERF4.2* (ON652753), *SlAP2a* (NP_001234452.1), *SlERF6* (NP_001266125.1), *MdERF1* (BAF43419.1), *MdERF2* (NP_001280848.1), *AdERF9* (ADJ67438.1), *MaERF11* (XP_009412068), *MdERF4* (NP_001306183.1), *EjERF11* (AKN10304.1), *PyTubulin* (XM_009376045.2), *PyActin* (JN684184), *PyERF3* (ASY06613.1), *PyMYB114* (ASY06612.1), *PybHLH3* (XP_048442703.1), *PyANS* (Pbr001543.2), *PyDFR* (Pbr020145.1), *PyUFGT* (Pbr039986.1), *Fv26S* (gene11892), *FvActin* (gene22626), *FvANS* (gene32347), *FvDFR* (gene15174), *FvUFGT* (gene12591), *SlTubulin* (Solyc08g006890), *SlActin* (Solyc03g078400), *SlERF4.1* (Solyc10g009110.1.1), *SlERF3* (XP_004249668.1), *SlMYB114* (XP_004237817.1), *SlANS* (Solyc08g080040), *SlDFR* (Solyc02g085020), *SlUFGT* (XM_004247965).

## Supplementary Material

kiad068_Supplementary_Data

## Supplemental data

The following materials are available in the online version of this article.

**Supplemental Figure S1**. Determination of total anthocyanin contents and analysis of anthocyanin biosynthesis-related gene expression patterns in ‘Jinzheng NO.1’ and ‘Starkrimson’ pears at 30, 60 and 90 DAFB.

**Supplemental Figure S2**. The phenotype of T2 generation tomato plants with *ERF4.1* overexpression and deletion mutation.

**Supplemental Figure S3**. Identification of *ERF4.1* deletion in T2 generation tomato mutants.

**Supplemental Figure S4**. Interaction validation of PyERF4.1/PyERF4.2 with PyMYB114 or PybHLH3.

**Supplemental Figure S5**. Validation of the effect of *PyMYB114* and *PybHLH3* overexpression on the transcriptional activity of *PyERF4.1*/*PyERF4.2*.

**Supplemental Figure S6**. The amino acid sequences of PyERF4.2 and PyERF4.2 after EAR motif mutation (PyERF4.2M; Met residues mutated to Leu residues), with the EAR motif in red lettering.

**Supplemental Table S1**. RT-qPCR primers used in this study.

**Supplemental Table S2**. The primers used for vector construction in this study.
