# Supplementary material for: ETHYLENE RESPONSE FACTORS 4.1/4.2 with an EAR motif repress anthocyanin biosynthesis in red-skinned pears
Source: Plant Physiol. 2023 Feb 3;192(3):1892–912. doi: 10.1093/plphys/kiad068 (PMC10315276; doi:10.1093/plphys/kiad068)
Supplement: kiad068_Supplementary_Data [file kiad068_supplementary_data.pdf]

Supplemental Figure S1

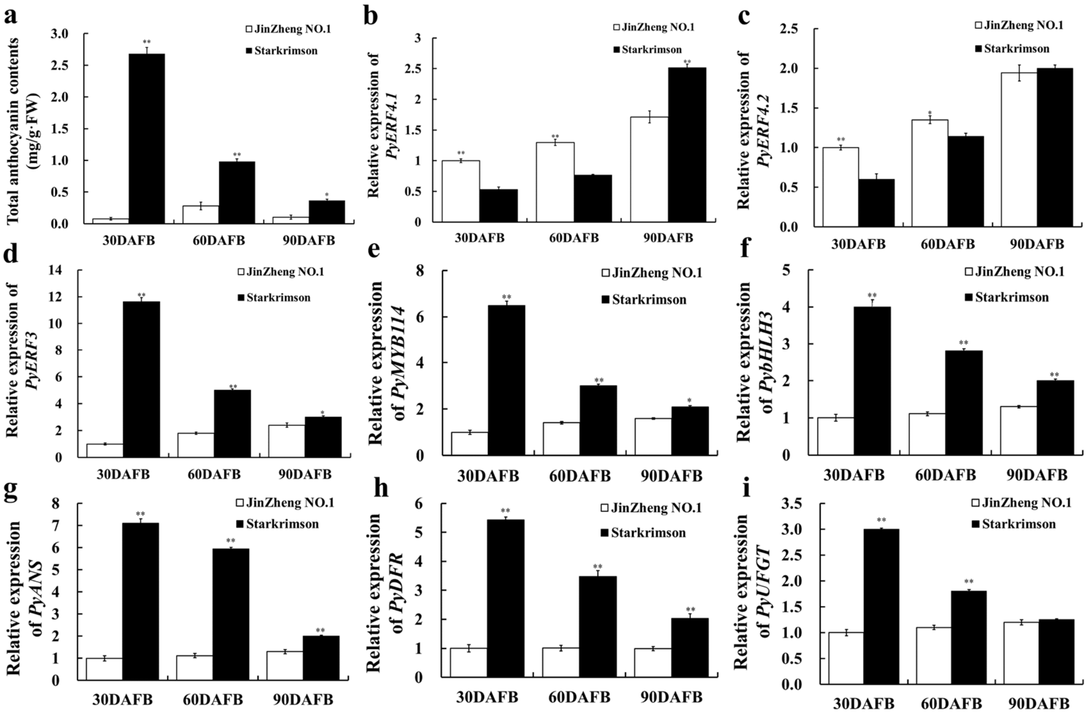

Supplemental Figure S1. Determination of total anthocyanin contents and analysis of anthocyanin biosynthesis-related gene expression patterns in 'JinZheng NO.1' and 'Starkrimson' pears at 30, 60 and 90 DAFB. (a) Determination of total anthocyanin contents. **(b-c)** Relative expression of *PyERF4.1* and *PyERF4.2*. **(d-f)** Relative expression of *PyERF3*, *PyMYB114* and *PybHLH3*. **(g-i)** Relative expression of *PyANS*, *PyDFR* and *PyUFGT*.

DAFB, days after full bloom Bars indicate mean values ± SD from three biological replicates. Student's *t*-test was used for statistical analysis, significance was indicated by asterisks * ($p < 0.05$) or ** ($p < 0.01$).

Supplemental Figure S2

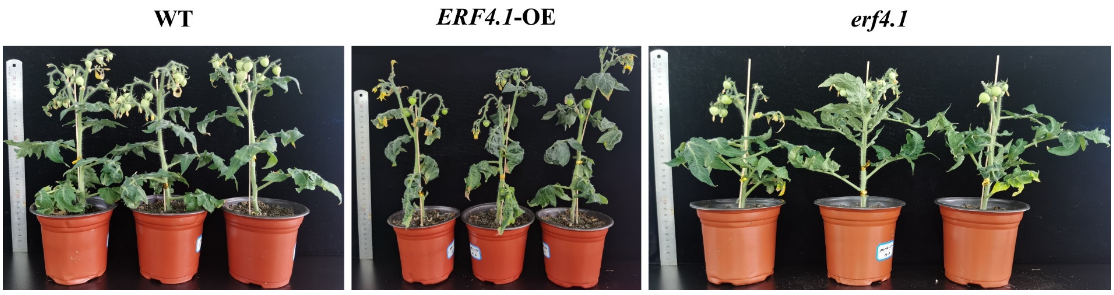

Supplemental Figure S2. The phenotype of T2 generation tomato plants with ERF4.1 overexpression and deletion mutation. WT, wild type; OE, overexpression; *erf4.1*, *ERF4.1* deletion mutant.

Supplemental Figure S3

a **>SlERF4.1**

MAPKEKIGAVTAMAMVNLNGISKEVHYRGVRKRPWGRYAAEIRDPGKKSRVWLGTFDTAEEAARAYDNAAREF
RGAKAKTNFPKLEMEKEEDLKFAVKNEINRSPSQTSTVESSSPVMVDSSSPLDLSLCGSIGGFNHHTVKFPSSGGG
FTGSVQAVNHMYYIEALARAGVIKLETNRKKTVDYLGGGDSDSSTVIDFMRVDVKSTTAGLNLDLNFPPPENM*

b **Target one** Sample: erf4.1.ab1

```
Anchor sequence: AAAGTAGGGTTTGGTTAGGT
Degenerate sequence: TGATTCATCATCGGA (Homozygous mutant)

Allele1: AAAGTAGGGTTTGGTTAGGTTGATTCATCATCGGA
Allele2: AAAGTAGGGTTTGGTTAGGTTGATTCATCATCGGA

Alignments of Allele1, Allele2, and reference sequence:
Allele1:   AAAGTAGGGTTTGGTTAGGTTGATTCATCATCGGAGTCCGAGTC (substitution)
Allele2:   AAAGTAGGGTTTGGTTAGGTTGATTCATCATCGGAGTCCGAGTC (substitution)
Reference: AAAGTAGGGTTTGGTTAGGTACTTTCGATACTGCGGAGGAGGCGGCTAGAGCTTATGATAACGCTGCTAGAGAATT
```

Target two Sample: erf4.1.ab1

```
Anchor sequence: AAAGTTAGAAACAAATCGGA
Degenerate sequence: CTCTGATTCATCAAC (Homozygous mutant)

Allele1: AAAGTTAGAAACAAATCGGACTCTGATTCATCAAC
Allele2: AAAGTTAGAAACAAATCGGACTCTGATTCATCAAC

Alignments of Allele1, Allele2, and reference sequence:
Allele1:      AAAGTTAGAAACAAATCGGA-------------------------------CTCTGATTCATCAAC
(deletion)
Allele2:      AAAGTTAGAAACAAATCGGA-------------------------------CTCTGATTCATCAAC
(deletion)
Reference: AAAGTTAGAAACAAATCGGAAGAAAACGGTAGATTACCTCGGTGGTGGTGACTCTGATTCATCAAC
```

c

```
SlERF4.1.protein   MAPKEKIGAVTAMAMVNLNGISKEVHYRGVRKRPWGRYAA   40
SlERF4.1M.protein  MAPKEKIGAVTAMAMVNLNGISKEVHYRGVRKRPWGRYAA   40
Consensus          mapkekigavtamamvnlngiskevhyrgvrkrpwgryaa

SlERF4.1.protein   EIRDPGKKSRVWLGTFDTAEEAARAYDNAAREFRGAKAKT   80
SlERF4.1M.protein  EIRDPGKKSRVWLG.FIIGVRVARAYDNAAREFRGAKAKT   79
Consensus          eirdpgkksrvwlg f       araydnaarefrgakakt

SlERF4.1.protein   NFPKLEMEKEEDLKFAVKNEINRSPGQTSTVESSSPVMVD  120
SlERF4.1M.protein  NFPKLEMEKEEDLKFAVKNEINRSPGQTSTVESSSPVMVD  119
Consensus          nfpklemekeedlkfavkneinrspgqtstvessspvmvd

SlERF4.1.protein   SSSPLDLSLCGSIGGFNHHTVKFPSSGGGFTGSVQAVNRM  160
SlERF4.1M.protein  SSSPLDLSLCGSIGGFNHHTVKFPSSGGGFTGSVQAVNRM  159
Consensus          ssspldlslcgsiggfnhhtvkfpssgggftgsvqavnrm

SlERF4.1.protein   YYIEALARAGVIKLEQIGRKRLDYLGGGDSDSSTVIDFMR  200
SlERF4.1M.protein  YYIEALARAGVIKLEQIGSDSS...........TVIDFMR  188
Consensus          yyiealaragvikleqig               tvidfmr

SlERF4.1.protein   VDVKSTTAGLNLDLNFPPPEN                     221
SlERF4.1M.protein  VDVKSTTAGLNLDLNFPPPEN                     209
Consensus          vdvksttaglnldlnfpppen
```

Supplemental Figure S3. Identification of *ERF4.1* deletion in T2 generation tomato mutants. (a) The protein sequence of SlERF4.1. The red marked section is the complete EAR motif. **(b)** Identification of two targets mutation types in T2 generation *ERF4.1* deletion tomato plants by DSDecodeM (http://skl.scau.edu.cn/dsdecode/). Short lines represent amino acid deletions. **(c)** The protein sequence of SlERF4.1 with

deletion mutation. M, mutation. The same amino acids were compared with dark blue markers. Different amino acids were compared with light blue markers. The dots represent amino acid deletions.

Supplemental Figure S4

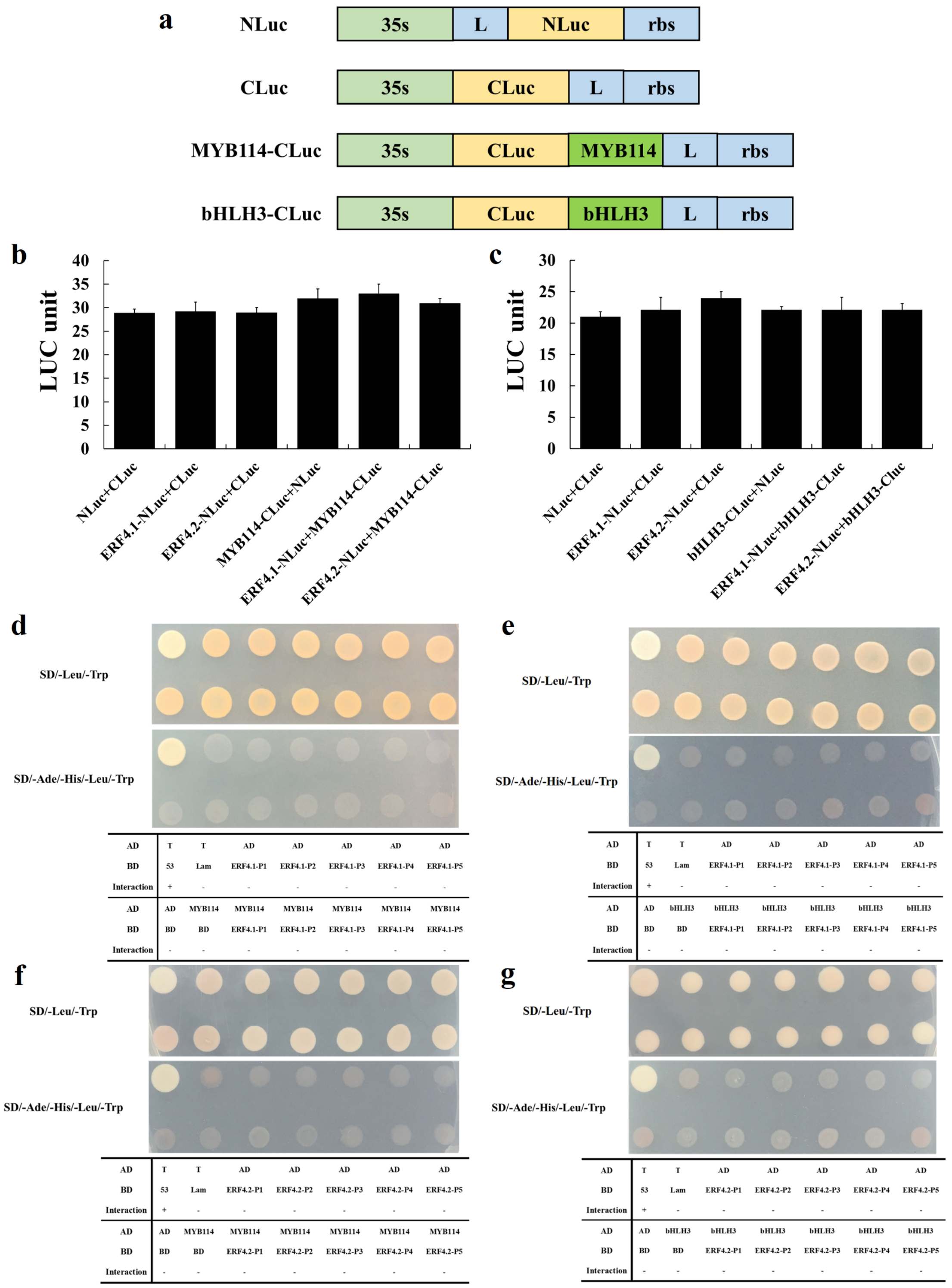

Supplemental Figure S4. Interaction validation of PyERF4.1/PyERF4.2 with PyMYB114 or PybHLH3. (a) Model of the NLuc, CLuc and NLuc/CLuc constructs. **(b-c)** Interaction validation of PyERF4.1/PyERF4.2 with PyMYB114 and PybHLH3

via firefly luciferase complementation assays. Bars indicate mean values ± SD from six biological replicates. **(d-e)** Interaction of PyERF4.1 with PyMYB114 and PybHLH3 verified by Y2H assays. **(f-g)** Interaction of PyERF4.2 with PyMYB114 and PybHLH3 verified by Y2H assays.

Statistical analysis was carried out with One-way ANOVA, and significance was marked with different letters ($p < 0.05$).

Supplemental Figure S5

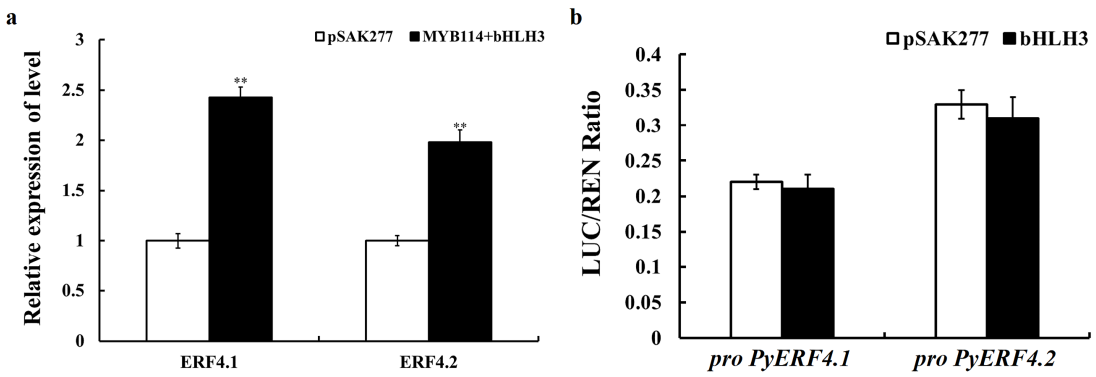

Supplemental Figure S5. Validation of the effect of *PyMYB114* and *PybHLH3* overexpression on the transcriptional activity of *PyERF4.1*/*PyERF4.2*. (a) Expression levels of *PyERF4.1* and *PyERF4.2* when *PyMYB114* and *PybHLH3* were transiently overexpressed. Bars indicate mean values ± SD from three biological replicates. **(b)** The dual-luciferase reporter assay in tobacco leaves verifies that PybHLH3 does not activate the *PyERF4.1* and *PyERF4.2* promoters.

Data are presented as means ± SD (n = 6). Statistical analysis was carried out with Student's t-test, and significance was marked with asterisks * ($p < 0.05$) or ** ($p < 0.01$).

Supplemental Figure S6

>PyERF4.2

MAPTTEKSPPKSGSEDPHPTSNETRFRGVRKRPWGRYAAEIRDPGKKTRVWLGTFDTAEEAACAYDKAAREFRG
GKAKTNFPTPTELHLDAVNIANMNNAAKLAATTNISNSPSSHSSTVESSSPPSPRPLDLTLKTPRLSTGGGYFTAAS
HQAQATATRPRPRSSTSSAPPATGGSISTSTFLPPQKSPKLPPPAAKDPPFFLSGASLFLFM*

>PyERF4.2M

MAPTTEKSPPKSGSEDPHPTSNETRFRGVRKRPWGRYAAEIRDPGKKTRVWLGTFDTAEEAACAYDKAAREFRG
GKAKTNFPTPTELHLDAVNIANMNNAAKLAATTNISNSPSSHSSTVESSSPPSPRPLDLTLKTPRLSTGGGYFTAAS
HQAQATATRPRPRSSTSSAPPATGGSISTSTFLPPQKSPKLPPPAAKDPPFFLSGASLFLFL*

Supplemental Figure S6. The amino acid sequences of PyERF4.2 and PyERF4.2 after EAR motif mutation (PyERF4.2M; Met residues mutated to Leu residues), with the EAR motif in red lettering.

Supplemental Table S1. RT-qPCR primers used in this study.

**Gene**	**Forward primer (5' to 3')**	**Reverse primer (5' to 3')**
PyTubulin	GGGCTTTGCTCCTCTTACTT	GATCAGCAGCACACATCATATTC
PyActin	TCCAGAAGAGCATCCAGTCC	GCCAGGTCCAAACGAAGG
PyERF4.1	AAGGCCAAGACCAACTTCC	CTGATTGTTGCTTCCGCTACTA
PyERF4.2	CATCCAACGAGACCCGATTC	TCTTCTTCCCGGGATCTCTAAT
PyERF3	CGGTGGAGTTACGTCTAGTTTC	GCAGGAGAAGCAGTTGAAGA
PyMYB114	GCCACATCCGTCATAAGACCTC	GCCACTCATGTGTAACCCTTC
PybHLH3	TTGTGGAGGGAAGTGGCGGT	AGCTCCCTAAGTGTTTGCATCAC
PyANS	GAGCAGAAGGAGAAGTAT	ACAGTGGAAGAAGTAGTC
PyDFR	GGTTTCATCGGCTCTTGGC	CCTTCTTCTGATTCGTGGGGT
PyUFGT	CCTTCCCTTTTGCCACTCA	CAGCCACATCGTACACCCTTA
Fv26S	AGCCTAACGCAGAGGTTCCAAA	GCAGCCCACATTGAAGGGTCTATA
FvActin	GCCAACCGTGAGAAGATG	TCCAGAGTCAAGAACAATACCAG
FvANS	GAAGTGCGTACCCAACTCCATCGT	ACCTTCTCCTTGTTGACGAGCCC
FvDFR	CACGATTCACGACATTGCGAAATT	GAACTCAAACCCCATCTCTTTCAGC
FvUFGT	CTAAGCAAAGGAAAGTTGAACGGAAT	TCCAACCGCAATGTGTTACAAA
SlTubulin	TAGAGCCTGGTACGATGGATAG	CAACTCAGCGCCTTCAGTATAA
SlActin	GGGATGGAGAAGTTTGGTGGTGG	CTTCGACCAAGGGA TGGTGTAGC
SlERF4.1	GCATTATAGAGGTGTAAGGAAGAGG	CCGCAGTATCGAAAGTACCTAAC
SlERF3	TGATGTCGGAATCGGCTAATC	CGAAGTCGTAACACCGCTAATA
SlMYB114	CCCATAAGAGCTGGTCTGAATAG	TTTCATCCGAAGCGAAGTCA
SlANS	GAACTAGCACTTGGCGTCGAA	TTGCAAGCCAGGCACCATA
SlDFR	AGTCCAAGGATCCAGAGAACGAAGTA	TGGACA TCAAGAGTTCCAGCAGAT
SlUFGT	CCAACAAGTTACAGCGAC	CAATGGGACACAAATCCTC

Supplemental Table S2. The primers used for vector construction in this study.

The purpose	The primers	The sequences of the primer (5' to 3')
For construction of recombinant vector of pSAK277	*PyERF4.1-Eco*R I-F	actagtggatccaaagaattcATGGCGCCGAGAGAGAAGA
	PykERF4.1-Xba I-R	tcattaaagcaggactctagaTCAAGCGAGCTCCGGTGG
	*PyERF4.2-Eco*R I-F	actagtggatccaaagaattcATGGCTCCGACGACGGAG
	PyERF4.2-Xba I-R	tcattaaagcaggactctagaTTACATAAAAAGGAACAAACTTGCACC
	*PyERF4.1*ΔE-*Eco*R I-F	actagtggatccaaagaattcATGGCGCCGAGAGAGAAGA
	*PyERF4.1*ΔE-*Xba* I-R	tcattaaagcaggactctagaGAACCCTCCGCCGCCGCG
	*PyERF4.2*ΔE-*Eco*R I-F	actagtggatccaaagaattcATGGCTCCGACGACGGAG
	*PyERF4.2*ΔE-*Xba* I-R	tcattaaagcaggactctagaACTTGCACCGGAAAGAAAAAAA
	*PyERF4.2*M-*Eco*R I-F	actagtggatccaaagaattcATGGCTCCGACGACGGAG
	*PyERF4.2*M-*Xba* I-R	tcattaaagcaggactctagaTTATAGAAAAAGGAACAAACTTGCACCGGA
	*PyERF3-Eco*R I-F	actagtggatccaaagaattcATGTTTTTGGGGTACAGTCGGG
	PyERF3-Xba I-R	tcattaaagcaggactctagaTCAACTGGATGAGGATGGATTGTTGC
	*PyMYB114-Eco*R I-F	actagtggatccaaagaattcATGAGGAAGGGTGCCTGG
	PyMYB114-Xba I-R	tcattaaagcaggactctagaCTAAATCTTAGTTATCTCTTCTTCTAGATTCCA
	*PybHLH3-Eco*R I-F	actagtggatccaaagaattcATGGCTGCACCGCCGCCAAG
	PybHLH3-Xba I-R	tcattaaagcaggactctagaTTAAGAGTCAGATTGGGGTATAATTTGATTTATC
For construction of recombinant vector for RNAi	*PyERF4.1*-RNAi-*Eco*R I -F	actagtggatccaaagaattcTGGTCGAGTCCTCGCCGT
	PyERF4.1-RNAi-*Xba* I-R	tcattaaagcaggactctagaAGGACGAGTCCGAGTCGCTC
	PyERF4.2-RNAi-*Eco*R I -F	actagtggatccaaagaattcTCCTCCTCCCCGCCGTCT
	PyERF4.2-RNAi-*Xba* I-R	tcattaaagcaggactctagaTTACATAAAAAGGAACAAACTTGCACC
For the cloning of promoter sequences and the construction of pGreen II 0800-LUC	*PyERF4.1-Hin*d III-F	gtcgacggtatcgataagcttCACTCATCCTTTTATTTTACAAATGATTT
	*PyERF4.1-Bam*H I-R	cgctctagaactagtggatccTATTACGTGGCATGTGTGGGTT
	*PyERF4.2-Hin*d III-F	gtcgacggtatcgataagcttAAAAGTTATAATAATTGTAGCCGTTGAAT
	*PyERF4.2-Bam*H I-R	cgctctagaactagtggatccTCTCTACAAATTTAATGCTGGTTTTGG
	*PyANS-Hin*d III-F	gtcgacggtatcgataagcttACAAATGCAAATTCACACACAAAA
	*PyANS-Bam*H I-R	cgctctagaactagtggatccTTTTGGAGCTGGCTTTCGAC
	*PyDFR-Hin*d III-F	gtcgacggtatcgataagcttAAATTTCCTAAAAAATATACTCGTGTTGA
	*PyDFR-Bam*H I-R	cgctctagaactagtggatccCTTATAAAATATTTATAGTGGGGGGCT
	*PyUFGT-Hin*d III-F	gtcgacggtatcgataagcttTACAAATATTCAGAGCATATATATCAATAGTAGTG
	*PyUFGT-Bam*H I-R	cgctctagaactagtggatccTGACTGACGGTTGCTATAGGTATTAA
	PyERF4.1-Nluc-*Bam*H I-F	cgagctcggtacccgggatccATGGCGCCGAGAGAGAAGAC

For construction of recombinant vector of Nluc/Cluc	*PyERF4.1*-Nluc-*Sal* I-R	cgcgtacgagatctggtcgacAGCGAGCTCCGGTGGTGGAGGAA
	PyERF4.2-Nluc-*Bam*H I-F	cgagctcggtacccgggatccATGGCTCCGACGACGGAGAA
	PyERF4.2-Nluc-*Sal* I-R	cgcgtacgagatctggtcgacCATAAAAAGGAACAAACTTGCACCGG
	PyERF4.2-Cluc-*Bam*H I-F	ccggggcggtacccgggatccATGGCTCCGACGACGGAGAA
	PyERF4.2-Cluc-*Sal* I-R	acgaaagctctgcaggtcgacCATAAAAAGGAACAAACTTGCACCGG
	PyERF3-Cluc-*Bam*H I-F	ccggggcggtacccgggatccATGTTTTTGGGGTACAGTCGGG
	PyERF3-Cluc-*Sal* I-R	acgaaagctctgcaggtcgacTCAACTGGATGAGGATGGATTG
	PyMYB114-Cluc-*Bam*H I-F	ccggggcggtacccgggatccATGAGGAAGGGTGCCTGGACTCAA
	PyMYB114-Cluc-*Sal* I-R	acgaaagctctgcaggtcgacCTAAATCTTAGTTATCTCTTCTTCTAG
For construction of recombinant vector of the yeast expression	*PyERF4.1*-P1-BK-*Nde* I-F	tcagaggaggacctgcatatgATGGCGCCGAGAGAGAAGA
	PyERF4.1-P1-BK-*Pst* I-R	ctagttatgcggccgctgcagGGCACCGCGGAACTCTATG
	PyERF4.1-P2-BK-*Nde* I-F	tcagaggaggacctgcatatgATGGCGCCGAGAGAGAAGA
	PyERF4.1-P2-BK-*Pst* I-R	ctagttatgcggccgctgcagAGCTCCGAATCCTGGAACCT
	PyERF4.1-P3-BK-*Nde* I-F	tcagaggaggacctgcatatgATGGCGCCGAGAGAGAAGA
	PyERF4.1-P3-BK-*Pst* I-R	ctagttatgcggccgctgcagTCAAGCGAGCTCCGGTGG
	PyERF4.1-P4-BK-*Nde* I-F	tcagaggaggacctgcatatgAAGGCCAAGACCAACTTCCC
	PyERF4.1-P4-BK-*Pst* I-R	ctagttatgcggccgctgcagTCAAGCGAGCTCCGGTGG
	PyERF4.1-P5-BK-*Nde* I-F	tcagaggaggacctgcatatgGTCATCGGAGTTCCGTCTTCC
	PyERF4.1-P5-BK-*Pst* I-R	ctagttatgcggccgctgcagTCAAGCGAGCTCCGGTGG
	*PyERF4.1*ΔE-BK-*Nde* I-F	tcagaggaggacctgcatatgATGGCGCCGAGAGAGAAGA
	*PyERF4.1*ΔE-BK-*Pst* I-R	ctagttatgcggccgctgcagGAACCCTCCGCCGCCGCG
	PyERF4.2-P1-BK-*Nde* I-F	tcagaggaggacctgcatatgATGGCTCCGACGACGGAG
	PyERF4.2-P1-BK-*Pst* I-R	ctagttatgcggccgctgcagGCCTCCGCGGAATTCACG
	PyERF4.2-P2-BK-*Nde* I-F	tcagaggaggacctgcatatgATGGCTCCGACGACGGAG
	PyERF4.2-P2-BK-*Pst* I-R	ctagttatgcggccgctgcagGCCACCGGTGGACAAGCG
	PyERF4.2-P3-BK-*Nde* I-F	tcagaggaggacctgcatatgATGGCTCCGACGACGGAG
	PyERF4.2-P3-BK-*Pst* I-R	ctagttatgcggccgctgcagTTACATAAAAAGGAACAAACTTGCACC
	PyERF4.2-P4-BK-*Nde* I-F	tcagaggaggacctgcatatgAAGGCGAAGACCAACTTCCC
	PyERF4.2-P4-BK-*Pst* I-R	ctagttatgcggccgctgcagTTACATAAAAAGGAACAAACTTGCACC
	PyERF4.2-P5-BK-*Nde* I-F	tcagaggaggacctgcatatgGGCTACTTCACCGCCGCG
	PyERF4.2-P5-BK-*Pst* I-R	ctagttatgcggccgctgcagTTACATAAAAAGGAACAAACTTGCACC
	*PyERF4.2*ΔE-BK-*Nde* I-F	tcagaggaggacctgcatatgATGGCTCCGACGACGGAG
	*PyERF4.2*ΔE-BK-*Pst* I-R	ctagttatgcggccgctgcagACTTGCACCGGAAAGAAAAAAA
	PyERF4.1-AD-*Eco*R I-F	gccatggaggccagtgaattcATGGCGCCGAGAGAGAAGA

	PyERF4.1-AD-*Xho* I-R	acgattcatctgcagctcgagTCAAGCGAGCTCCGGTGG
	PyERF3-AD-*Eco*R I-F	gccatggaggccagtgaattcATGTTTTTGGGGTACAGTCGGG
	PyERF3-AD-*Xho* I-R	acgattcatctgcagctcgagTCAACTGGATGAGGATGGATTGT
	PyMYB114-AD-*Eco*R I-F	gccatggaggccagtgaattcATGAGGAAGGGTGCCTGGAC
	PyMYB114-AD-*Xho* I-R	acgattcatctgcagctcgagCTAAATCTTAGTTATCTCTTCTTCTAGATTCCA
	PybHLH3-AD-*Eco*R I-F	gccatggaggccagtgaattcATGGCTGCACCGCCGCCA
	PybHLH3-AD-*Xho* I-R	acgattcatctgcagctcgagTTAAGAGTCAGATTGGGGTATAATTTG
	PyHDA9-AD-*Eco*R I-F	gccatggaggccagtgaattcATGCCTTCAAAGGATAAAATCGC
	PyHDA9-AD-*Xho* I-R	acgattcatctgcagctcgagTTACATATCTTCCATGTGATCGTTATCG
	PyERF4.1-S1-pAbAi-*Hin*d III-F	aaatgatgaattgaaaagcttATGCTGACGAAGGAGGGGA
	PyERF4.1-S1-pAbAi-*Xho* I-R	atacagagcacatgcctcgagTTCATTTTTTAATTCTTAATGTAATTTTTAATT
	PyERF4.2-S1-pAbAi-*Hin*d III-F	aaatgatgaattgaaaagcttAAAAGTTATAATAATTGTAGCCGTTGAAT
	PyERF4.2-S1-pAbAi-*Xho* I-R	atacagagcacatgcctcgagAGCCCTACACATACGAAACCGG
	PyERF4.2-S2-pAbAi-*Hin*d III-F	aaatgatgaattgaaaagcttGCAATAGGTGGTTAAGCTGAGGC
	PyERF4.2-S2-pAbAi-*Xho* I-R	atacagagcacatgcctcgagGTAAGAAGGGTATTGGTTGCATCTAT
	PyERF4.2-S3-pAbAi-*Hin*d III-F	aaatgatgaattgaaaagcttCGCCAAACTTAATAACAACATATGAC
	PyERF4.2-S3-pAbAi-*Xho* I-R	atacagagcacatgcctcgagGGGTCTTTCGGAGCTTGATTT
For construction of recombinant vector for pull-down assay	*PyERF4.1*-MBP-pMAL-*Bam*H I-F	cgcgatatcgtcgacggatccATGGCGCCGAGAGAGAAGA
	PyERF4.1-MBP-pMAL-*Hin*d III-R	ctttcgttttatttgaagcttTCAAGCGAGCTCCGGTGG
	PyERF4.2-MBP-pMAL-*Bam*H I-F	aggatttcagaattcggatccATGGCTCCGACGACGGAG
	PyERF4.2-MBP-pMAL-*Hin*d III-R	acgacggccagtgccaagcttTTACATAAAAAGGAACAAACTTGCACC
	PyERF3-HIS-pCold-*Bam*H I-F	ctcggtaccctcgagggatccATGTTTTTGGGGTACAGTCGGG
	PyERF3-HIS-pCold-*Xba* I-R	agcagagattacctatctagaTCAACTGGATGAGGATGGATTGT
For construction of recombinant vector of pCAMBIA1300-221-35S-GUS and CRISPR-Cas9	*PyERF4.1*-OE-*Bam*H I-F	acgggggactctagaggatccATGGCGCCGAGAGAGAAGA
	PyERF4.1-OE-*Sac* I-R	cgatcggggaaattcgagctcTCAAGCGAGCTCCGGTGG
	PyERF4.2-OE-*Bam*H I-F	acgggggactctagaggatccATGGCTCCGACGACGGAG
	PyERF4.2-OE-*Sac* I-R	cgatcggggaaattcgagctcTTACATAAAAAGGAACAAACTTGCACC
	SlERF4.1-Cas9-F1	GTCAGGTACTTTCGATACTGCGG
	SlERF4.1-Cas9-R1	AAACCCGCAGTATCGAAAGTACC
	SlERF4.1-Cas9-F2	GTCAAAACGGTAGATTACCTCGG
	SlERF4.1-Cas9-R2	AAACCCGAGGTAATCTACCGTTT
	SP-DL	GTCGTGCTCCACATGTTG
	SP-R	CCCGACATAGATGCAATAACTTC
